# Phosphoproteomic Insights into TRPV4–AMPK Signaling Axis and Barrier Regulation in the Choroid Plexus Epithelium

**DOI:** 10.3390/ijms27188305

**Published:** 2026-09-18

**Authors:** Gowthami Mahendran, Maryam Torabi, Bonnie L Blazer-Yost

**Affiliations:** Hydrocephalus Research Center, Department of Biology, Indiana University, Indianapolis, IN 46202, USA; gmahendr@iu.edu (G.M.); mtorabi@iu.edu (M.T.)

**Keywords:** phosphorylation, AMPK, TRPV4, choroid plexus, hydrocephalus, epithelial barrier integrity

## Abstract

Hydrocephalus is characterized by the abnormal accumulation of cerebrospinal fluid (CSF) due to disrupted secretion, circulation, or reabsorption. CSF homeostasis is regulated by ion and water channels on choroid plexus epithelial (CPe) cells, including the mechanosensitive cation channel, which is transient receptor potential vanilloid 4 (TRPV4). Although TRPV4 antagonism prevents hydrocephalus progression in rats, how TRPV4 activity modulates CSF production remains unclear. Because TRPV4 function is phosphorylation-dependent, we examined its activation (GSK1016790A) and inhibition (RN1734) and the downstream signaling alterations in the human choroid plexus papilloma cell line (HIBCPP) using phosphoproteomic mass spectrometry. Our phosphoproteomic analysis revealed significant changes in kinases and tight junction (TJ) proteins regulating epithelial barrier integrity. TRPV4 activation altered the phosphorylation of TJ proteins, including Zonula Occludens-1 (ZO-1) and Claudin-7 (CLDN7), as well as AMP-activated protein kinase (AMPK), with a notable increase in the phosphorylation of the AMPK inhibitory site (Ser496). Using electrophysiological techniques, we showed that AMPK had a more profound effect on epithelial barrier function than on net transepithelial electrolyte ion flux. The inhibition of AMPK-associated inhibitory phosphorylation prior to TRPV4 activation increased barrier tightness, and these permeability changes were mirrored by changes in ZO-1 continuity within junctional complexes. Furthermore, the pharmacological modulation of AMPK-associated signaling altered TRPV4-stimulated epithelial conductance, while metformin treatment also reduced TRPV4-stimulated conductance under the experimental conditions used. These findings suggest an interaction between TRPV4 signaling, AMPK phosphorylation, and epithelial barrier regulation that warrants further mechanistic investigation. Overall, these findings provide insight into signaling pathways associated with the TRPV4-dependent regulation of choroid plexus epithelial barrier properties and provide a basis for the further investigation of their relevance to CSF homeostasis and hydrocephalus.

## 1. Introduction

The choroid plexus epithelium (CPe), present in the brain ventricles, is a specialized barrier epithelial monolayer surrounding a fenestrated capillary network and serves as the primary site of CSF production [1]. CSF plays a critical role in maintaining intracranial pressure and central nervous system (CNS) homeostasis. The CPe forms the blood–CSF barrier (BCSFB) through tight junction (TJ) complexes between adjacent epithelial cells, and thereby tightly regulates the transepithelial movement of ions and water to control CSF volume [1,2,3]. The disruption of CPe function or barrier integrity can alter CSF dynamics and contribute to neurological disorders such as hydrocephalus, which is a multifaceted neurological condition characterized by the abnormal accumulation of CSF within the brain ventricles and increased intracranial pressure [4,5]. This excessive fluid buildup leads to ventricular enlargement, disrupting delicate intracranial dynamics, impairing normal brain function and giving rise to a wide spectrum of neurological consequences, including cognitive and motor impairments as well as visual and gait disturbances [6,7,8,9]. Emerging evidence from experimental models of hydrocephalus indicates that alterations in epithelial barrier integrity and ion transport at the CPe contribute to these pathological changes, underscoring the need to elucidate the molecular mechanisms governing these processes [1,2,10,11,12].

The CPe barrier function is maintained by highly specialized TJ complexes that form the structural basis of the BCSFB. These junctions are composed of transmembrane proteins, including claudins, occludins, and junctional adhesion molecules, which assemble into continuous TJ strands that tightly regulate the paracellular movement of ions across the cells, defining a selective permeability of the epithelium [13]. The cytoplasmic domains of these TJ proteins are anchored to the actin cytoskeleton through scaffolding proteins of the zonula occludens (ZO) family, particularly ZO-1, which organize junctional architecture and integrate signaling pathways [14]. These CPe TJs are dynamic structures that can respond to intracellular signaling cascades, post-translational modifications such as phosphorylation, and mechanical forces, regulating epithelial permeability. Importantly, TJs also preserve epithelial polarity to ensure the proper localization and function of ion transporters that drive CSF secretion. The disruption of TJ organization by inflammation, oxidative stress, infection, or altered signaling can increase epithelial permeability, leading to abnormal ion fluxes and dysregulated CSF production [15]. However, the molecular sensors and signaling mechanisms controlling TJ remodeling in the CPe remain poorly understood.

One candidate mediator identified in the apical membrane of CPe that regulates CSF production is the transient receptor potential vanilloid 4 (TRPV4) [10,16], which is a mechanosensitive, nonselective cation-permeable ion channel that is activated by osmotic stress and mechanical stimuli and, when activated, results in an increase in intracellular Ca^2+^ [17]. As a mechanosensitive cation channel, TRPV4 has been shown to regulate cytoskeletal remodeling and intercellular junction organization in epithelial and endothelial cells [18,19,20]. TRPV4 links mechanical or osmotic stimuli to intracellular calcium signaling to coordinate the movement of ions and water into and out of the cells to maintain the CSF dynamics [10]. Studies show that targeting TRPV4 pharmacologically in the choroid plexus may attenuate CSF overproduction and ventricular enlargement in an experimental rat hydrocephalus model, highlighting its critical role in maintaining CSF homeostasis [16].

To study the role of CPe in CSF regulation, the HIBCPP cell line, derived from a choroid plexus papilloma of a human female, is a good epithelial cell model that mimics the in vitro characteristics of BCSFB [10,21,22]. HIBCPP cells retain key epithelial characteristics, including membrane polarity, TJ assembly, energy-sensing kinases, and the expression of transporters and water channel proteins to maintain CSF production and homeostasis in response to metabolic and mechanical stimuli. Studies have demonstrated the expression and polarized localization of TJ proteins, including ZO proteins, claudins, and occludins, as well as ion transporters such as TRPV4, the Na^+^/K^+^-ATPase α1 and β2 subunits, the Na^+^-K^+^-Cl^−^ cotransporter 1 (NKCC1), the sodium–bicarbonate cotransporter (NBCe2), and volume-regulated anion channels (VRAC) at the apical membrane, and the Na^+^/HCO_3_^−^ cotransporter (NBCe1) and anion exchanger 2 (AE2) at the basolateral membrane of HIBCPP cells [10]. Moreover, an electrophysiological study on HIBCPP cells by Hulme et al. reported a rapid multiphasic increase in short-circuit current (indicative of the net electrogenic ion movement across the cell) and conductance (a measure of transepithelial permeability) upon TRPV4 activation with the agonist (GSK1016790A). This study also demonstrated enhanced apical fluid secretion upon TRPV4 channel activation by GSK1016790A. However, the functional alterations induced after TRPV4 activation did not compromise the spatial organization of junctional proteins such as ZO-1, suggesting that the observed increase in permeability occurs independently of junctional complex disassembly [10]. Despite this evidence, the precise molecular pathways by which TRPV4 activity modulates TJ organization and epithelial permeability in the choroid plexus remain unclear.

TRPV4-mediated intracellular calcium signaling can activate a variety of kinase pathways and effectors (phospholipase C, protein kinase C, phosphatidylinositol 3-kinase (PI3K)), suggesting that channel-mediated modulation may occur through phosphorylation-dependent mechanisms [10,23,24]. One such kinase that our phosphoproteomics study discovered was AMP-activated protein kinase (AMPK), which is a heterotrimeric serine/threonine kinase that functions as a cellular energy sensor and metabolism controller that integrates metabolic and mechanical signals to regulate epithelial polarity, cytoskeletal dynamics, and junctional stability [25]. AMPK activity is regulated by site-specific phosphorylation events [26,27]. Phosphorylation of threonine 172 (Thr172) in the activation loop of the catalytic α1-subunit is essential for AMPK activation and is typically mediated by upstream kinases such as liver kinase B1 (LKB1) or calcium/calmodulin-dependent protein kinase kinase β (CaMKKβ) [26,27] (Figure S1). In contrast, phosphorylation at Ser496 has been reported to negatively regulate AMPK activation in specific cellular contexts, including through interference with Thr172 phosphorylation. Compared with the well-established role of Thr172 phosphorylation in AMPK activation, the physiological significance and context-dependent regulation of Ser496 remain less well-characterized. Phosphorylation of AMPKα1 at Ser496 has been reported to negatively regulate AMPK activity by limiting activating phosphorylation at Thr172. Ser496 phosphorylation can be mediated by upstream kinases, including protein kinase A (PKA), and may therefore provide an additional regulatory mechanism for modulating AMPK signaling [28,29] (Figure S1). Note that this inhibitory residue is numbered inconsistently across the literature depending on the isoform and numbering convention (e.g., Ser485, Ser487, or Ser496). Throughout this manuscript, we use Ser496, corresponding to the canonical UniProt Q13131 sequence used for our mass-spectrometry database search.

In epithelial cells, active AMPK (phosphorylation at Thr172) has been shown to stabilize TJs and adherens junctions under stress conditions, whereas inhibitory phosphorylation at sites like Ser496 (inactive AMPK) can promote junctional disassembly and increase paracellular permeability [30]. These dual regulatory mechanisms illustrate that modulation of AMPK phosphorylation could serve as a prime link between mechanosensitive signals, such as those initiated by TRPV4 activation, and dynamic control of epithelial barrier function. While AMPK has been implicated in TJ regulation in other epithelial systems [31], its potential role as a downstream effector of TRPV4 signaling in the choroid plexus remains unexplored.

In this study, we investigated phosphorylation-dependent signaling pathways downstream of TRPV4 activation in HIBCPP cells [22,32], with a particular emphasis on mechanisms governing TJ organization and epithelial permeability. HIBCPP cells were treated with a TRPV4 agonist (GSK1016790A) or antagonist (RN1734) followed by phosphoproteomic analyses to identify downstream signaling and metabolic pathways altered upon TRPV4 modulation. Notably, our study revealed candidate phosphorylation changes in kinases and TJ proteins involved in maintaining epithelial barrier integrity. Specifically, treatment with a TRPV4 antagonist followed by agonist exposure induced notable changes in ZO-1, whereas agonist treatment alone altered ZO-1, AMPK, and Claudin-7. TRPV4 activation increased inhibitory phosphorylation of AMPK at Ser496, suggesting an association between TRPV4 activation, AMPK phosphorylation, and epithelial barrier regulation. Our findings underscore the importance of AMPK-regulated pathways in epithelial barrier regulation. Elucidating these mechanisms will advance our understanding of how CPe function contributes to CSF homeostasis and how its dysregulation may underlie the pathophysiology of hydrocephalus.

## 2. Results

### 2.1. TRPV4-Dependent Changes Modulate the Phosphoproteome Network in HIBCPP Cells

**Figure 1:**
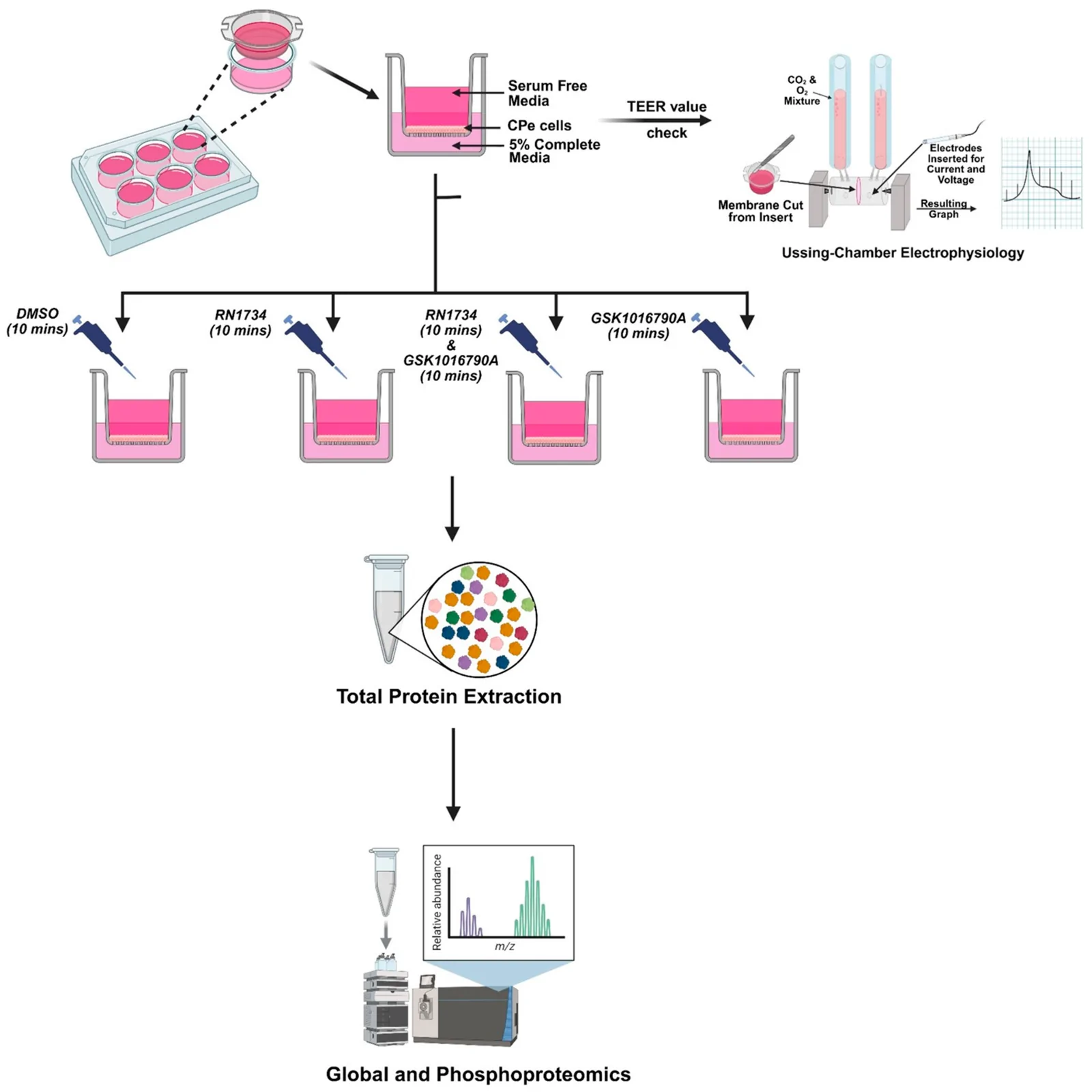
Schematic of experimental setup and drug treatment conditions in HIBCPP cells: Cells were grown on 6-well Transwell inserts with DMEM serum-free media on the apical side and DMEM complete media on the basal side. Transepithelial electrical resistance (TEER) was measured prior to downstream experiments to confirm barrier integrity of the epithelial cells. For mass spectrometry analysis, cells were treated under four conditions: DMSO for 10 min, GSK1016790A for 10 min, RN1734 for 10 min followed by GSK1016790A for 10 min, or RN1734 alone for 10 min, followed by total protein extraction for mass spectrometry. The figure was created using BioRender.

To investigate whether alterations to TRPV4 activity trigger rapid downstream phosphorylation events, we utilized a well-characterized in vitro model of human choroid plexus epithelial cells, HIBCPP. Global proteomics and phosphoproteomics mass spectrometry analyses were performed following short-term (10 min) pharmacological manipulation of TRPV4 with TRPV4 agonist (GSK1016790A) and TRPV4 antagonist (RN1734). The 10 min time frame was chosen to correlate with observed TRPV4-stimulated functional effects on transepithelial electrolyte and fluid movement [10]. Three experimental conditions were examined in this study: (1) TRPV4 activation (15 nM GSK1016790A vs. DMSO diluent), (2) TRPV4 inhibition followed by activation (25 µM RN1734 pretreatment + 15 nM GSK1016790A vs. DMSO), and (3) TRPV4 inhibition (25 µM RN1734 vs. DMSO) (Figure 1). The RN1734 + GSK1016790A condition was included to determine whether antagonist pretreatment alters phosphorylation responses induced by TRPV4 activation. Previous studies in HIBCPP cells have demonstrated that RN1734 substantially blocks TRPV4-mediated changes in transepithelial electrolyte flux and conductance following GSK1016790A stimulation [10]. The integrity of HIBCPP cell monolayers was assessed before their use in experiments using Ussing-style electrophysiology to measure net ion flux and transepithelial permeability. This ensured that each cell passage used in this study exhibited appropriately high transepithelial resistance and a secretory phenotype.

**Figure 2:**
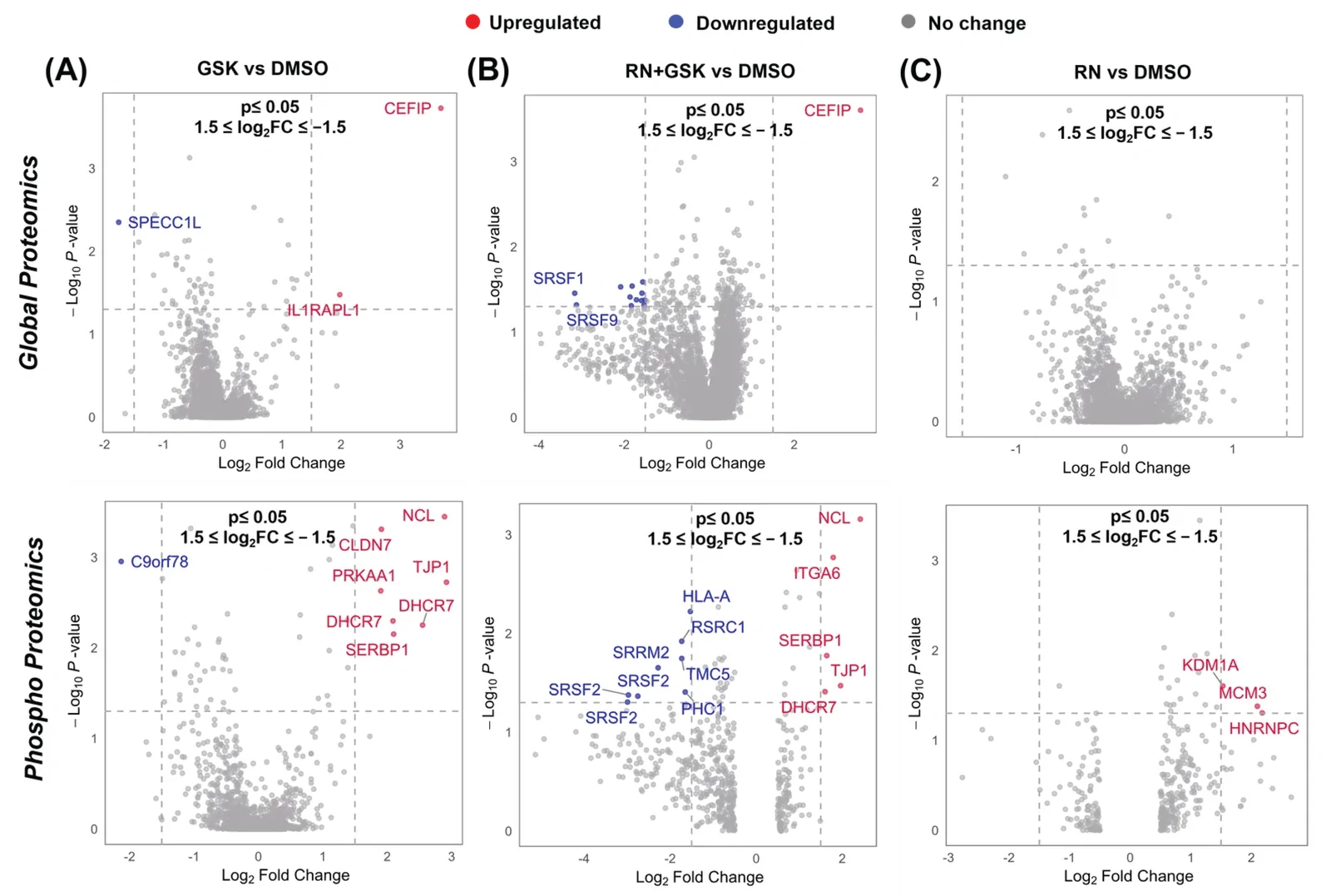
Differential protein abundance and phosphorylation changes identified by global proteomic (**top**) and phosphoproteomic (**bottom**) analyses: Volcano plots illustrate candidate proteins/phosphopeptides meeting the exploratory thresholds of nominal *p* < 0.05 and |log_2_ fold change| ≥ 1.5 (indicated by dashed lines), across three experimental conditions compared with control: (**A**) TRPV4 agonist (GSK1016790A) vs. DMSO, (**B**) TRPV4 antagonist (RN1734) + TRPV4 agonist (GSK1016790A) vs. DMSO, and (**C**) TRPV4 antagonist (RN1734) vs. DMSO. Upregulated, downregulated, and non-significant proteins are shown in red, blue, and gray, respectively. Mass spectrometry analyses were performed using biological triplicates for all conditions (*n* = 3).

As expected during this short time period, global proteomics analysis revealed no significant changes to the overall protein abundance across any treatment groups within the defined log_2_ fold change and −log_10_ *p*-value thresholds (Figure 2, top panel). In contrast, phosphoproteomics analysis identified treatment-specific changes in protein phosphorylation, highlighting acute phosphorylation events as a significant downstream response that alter TRPV4 activity (Figure 2, bottom panel).

In the first experimental group, in which TRPV4 was activated (GSK1016790A vs. DMSO), seven phosphoproteomic events met the predefined exploratory thresholds of nominal *p* < 0.05 and |log_2_ fold change| ≥ 1.5, with six showing increased phosphorylation (NCL, CLDN7, PRKAA1, TJP1, DHCR7, and SERBP1) and one showing decreased phosphorylation (C9orf78) (Figure 2A). Particularly, these proteins are associated with diverse functions including TJ organization in epithelial cells (CLDN7 or claudin-7 [33], TJP1 or ZO-1 [34,35]), cellular energy homeostasis (PRKAA1 or AMPK [36]), and RNA binding proteins (NCL [37], SERBP1 [38]), highlighting that TRPV4 activation rapidly influences epithelial barrier-associated signaling and metabolic regulation. In the second experimental group where the cells are first treated with a TRPV4 antagonist and then the agonist (RN1734 + GSK1016790A vs. DMSO), eleven phosphoproteomic events met the exploratory thresholds showing increase in expression (NCL, ITGA6, SERBP1, TJP1, DHCR7) and six of them decreased in expression (HLA-A, RSRC1, TMC5, PHC1, SRRM2, and SRSF2) (Figure 2B). However, these include multiple phosphorylation events mapping to the same protein and therefore should not be interpreted as unique phosphoproteins. In the third experimental group, the TRPV4 antagonist alone (RN1734 vs. DMSO), three phosphoproteomic events met the exploratory thresholds, showing increased expression (KDM1A, MCM3, and HNRNPC) (Figure 2C).

**Figure 3:**
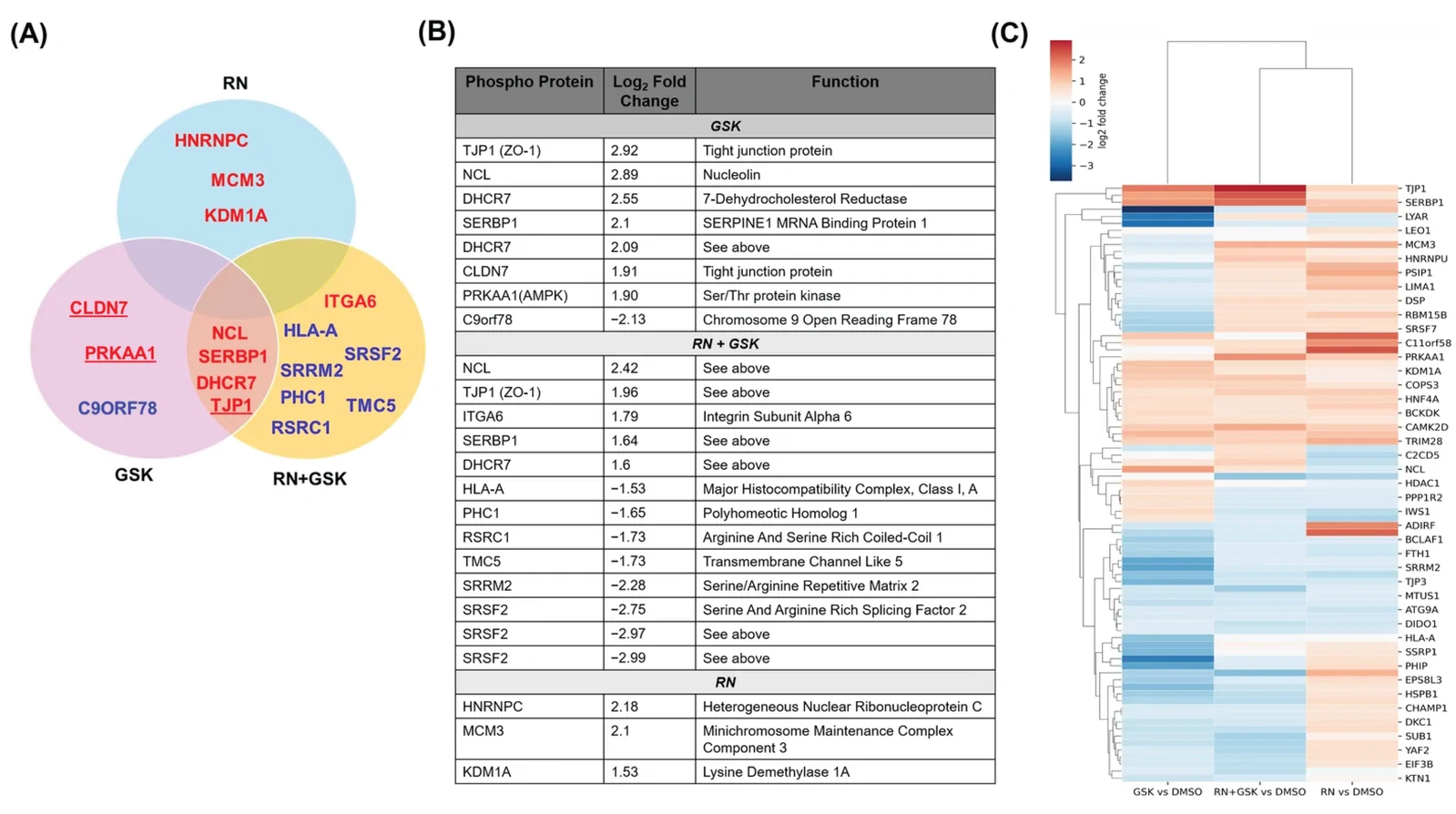
Distinct and shared phosphoproteomic events identified across TRPV4 modulation conditions: (**A**) Venn diagram showing the overlap of candidate phosphorylation changes among GSK1016790A, RN1734, and combined treatment conditions (RN1734+ GSK1016790A) relative to DMSO control. Proteins unique to each condition and those commonly regulated across multiple conditions are indicated, with upregulated proteins shown in red and downregulated proteins shown in blue. Phosphoproteins selected for further study are underlined. (**B**) Table summarizing selected significantly regulated proteins for each comparison, including log_2_ fold change values and their annotated biological functions. (**C**) Hierarchical clustering heatmap of candidate phosphorylation features across all experimental conditions. Phosphoproteomic abundance values are Z-score normalized and color-coded, with dark red indicating higher phosphoproteomic abundance and blue indicating lower phosphoproteomic abundance relative to DMSO control. Columns represent different experimental conditions (GSK1016790A vs. DMSO, RN1734 + GSK1016790A vs. DMSO, and RN1734 vs. DMSO), and rows represent individual proteins. Heatmap intensities represent averaged measurements from biological triplicates (*n* = 3).

Of the phosphoproteins identified in the GSK1016790A vs. DMSO comparison, TJP1 (ZO-1), CLDN7 (claudin-7), and PRKAA1 (AMPK) were prioritized as the top functionally relevant targets for downstream study, based on their established roles in epithelial barrier regulation, membrane organization, and cellular energy sensing (Figure 3A). The table in Figure 3B summarizes the phosphoproteins, their corresponding log_2_ fold changes, and their key functions identified in this study. Further, the heatmap visually represents the phosphorylation patterns of the key phosphoproteins identified across the three experimental groups. Hierarchical clustering highlights distinct phosphorylation signatures corresponding to each experimental condition (Figure 3C). The clustering pattern underscores the differential impact of TRPV4 modulation on phosphorylation networks, supporting the concept that TRPV4 activity coordinates a multifaceted phosphoproteomic response in CPe cells.

ZO-1, predominantly located in the cytoplasm, is a critical scaffolding protein. Phosphorylation of ZO-1 promotes its recruitment from the cytoplasm to TJs, enhancing subcellular localization at the junctional membrane [39]. This redistribution strengthens TJ assembly and barrier integrity, which is critical for maintaining CPe function [35]. Calcium-dependent kinases, including AMP-activated protein kinase (AMPK), are known to regulate ZO-1 phosphorylation indirectly through intermediate proteins such as afadin and cingulin, thereby linking intracellular calcium flux to TJ remodeling [40,41,42]. Additionally, CLDN7, a transmembrane TJ protein belonging to the claudin family, plays an essential role in maintaining epithelial barrier selectivity and junctional stability [43]. Phosphorylation of claudins (claudin-1, -3, -4, -5, -7, and -16) has been reported to influence their trafficking, membrane localization, and incorporation into TJ strands. Unlike classical barrier-forming claudins, CLDN7 has a role in maintaining epithelial architecture, cell–matrix interactions, and overall tissue integrity [44]. It has also been shown to interact with integrins and other membrane proteins, linking TJ organization to cell adhesion and signaling pathways [45]. As a result, its function extends beyond barrier formation to include regulation of epithelial homeostasis and structural stability.

Next, another crucial phosphoprotein detected in the GSK1016790A vs. DMSO experimental condition, PRKAA1, the catalytic α1 subunit of AMPK, represents a key metabolic sensor that integrates cellular energy status with epithelial barrier regulation. AMPK activation has been shown to promote TJ assembly and barrier integrity through phosphorylation of junctional proteins, including ZO-1, and through regulation of actin cytoskeletal dynamics [40]. The phosphorylation of AMPK following TRPV4 activation underscores a functional coupling between TRPV4-mediated calcium signaling and AMPK-dependent metabolic pathways.

**Figure 4:**
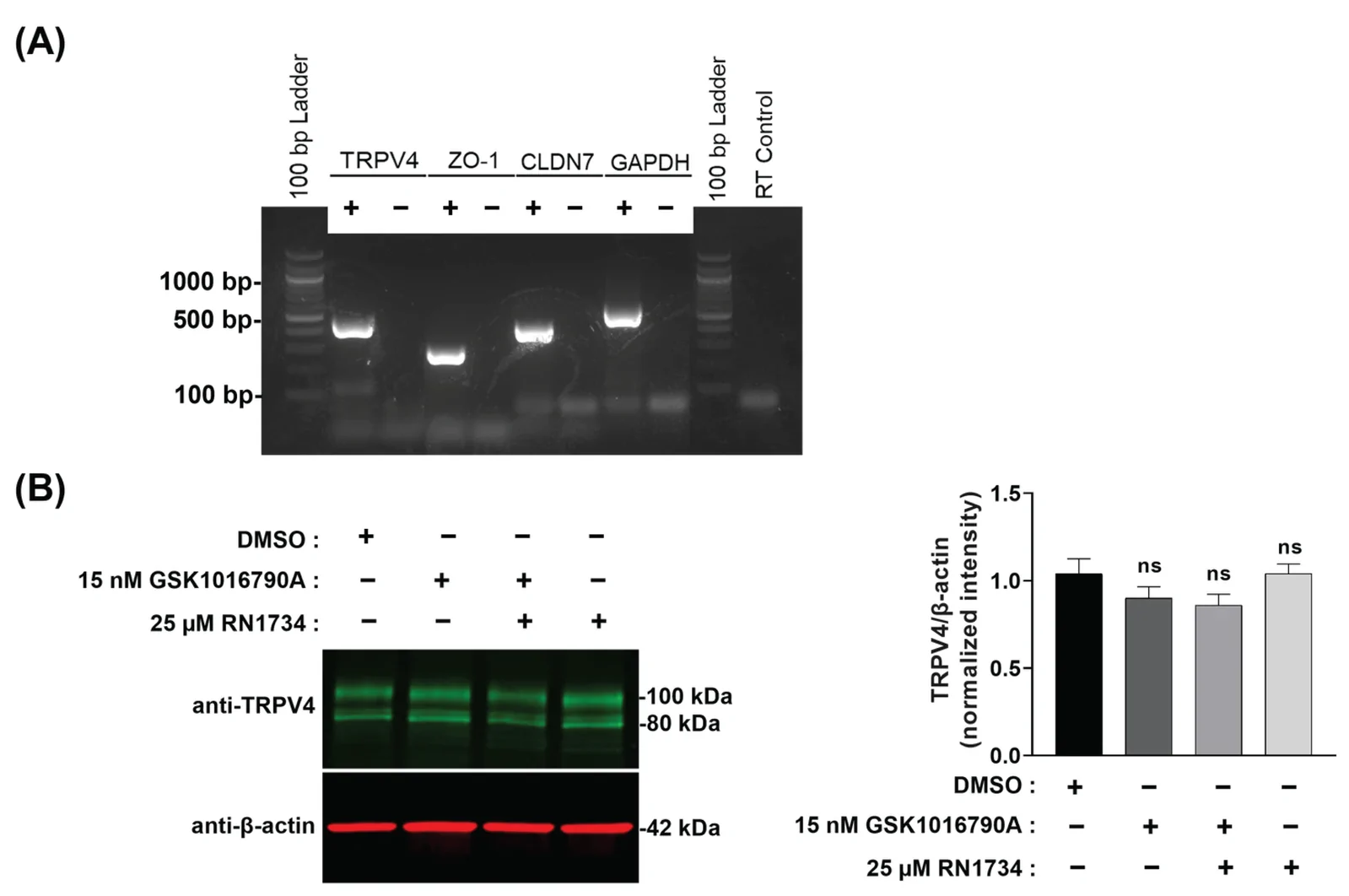
Confirmation of transporter and TJ protein expression in HIBCPP cells: (**A**) RT-PCR analysis of the transcripts for *TRPV4*, *ZO-1*, and *CLDN7*. RT (reverse transcriptase)-negative controls were included to confirm the absence of genomic DNA contamination, and no-template controls (indicated by “–” under each gene) were included to verify the specificity of amplification. PCR products were visualized on gels stained with SYBR Safe. GAPDH was used as a positive control (**B**) TRPV4 protein expression was compared across the indicated experimental groups. Western blot analysis revealed two bands at approximately 100 kDa and 80 kDa. Band intensities were normalized to the housekeeping protein β-actin. Quantification of the bands is shown on the right. The quantification represents the combined signal intensity of the approximately 100 kDa and 80 kDa TRPV4-immunoreactive bands, normalized to β-actin. Data are presented as mean ± standard deviation from three independent biological replicates (*n* = 3). Statistical significance was assessed using a two-tailed unpaired Student’s *t*-test; no detectable change in TRPV4 protein abundance was observed under the conditions tested relative to the DMSO control group (^ns^ *p* > 0.1234).

To further validate our phosphoproteomic findings, we examined the gene expression of selected TJ proteins including ZO-1, CLDN7, as well as the TRPV4 ion channel in the HIBCPP cells. Reverse transcription PCR (RT-PCR) analysis confirmed the expression of *ZO-1*, *CLDN7*, and *TRPV4* transcripts in HIBCPP cells (Figure 4A), verifying that these proteins identified in the phosphoproteomic analysis are transcriptionally expressed in this cell model.

### 2.2. TRPV4 Activation Increases Apical TRPV4 Intensity Without Altering Total Protein Abundance

Global TRPV4 protein levels in CPe cells were analyzed following 10 min pretreatment with TRPV4 agonist and antagonist (DMSO for 10 min, 15 nM GSK1016790A for 10 min, 25 µM RN1734 for 10 min + 15 nM GSK1016790A for 10 min, and 25 µM RN1734 for 10 min). No significant changes in TRPV4 expression were observed in any experimental group (Figure 4B), consistent with the expectation that short-term modulation of TRPV4 activity does not alter overall protein abundance of TRPV4.

**Figure 5:**
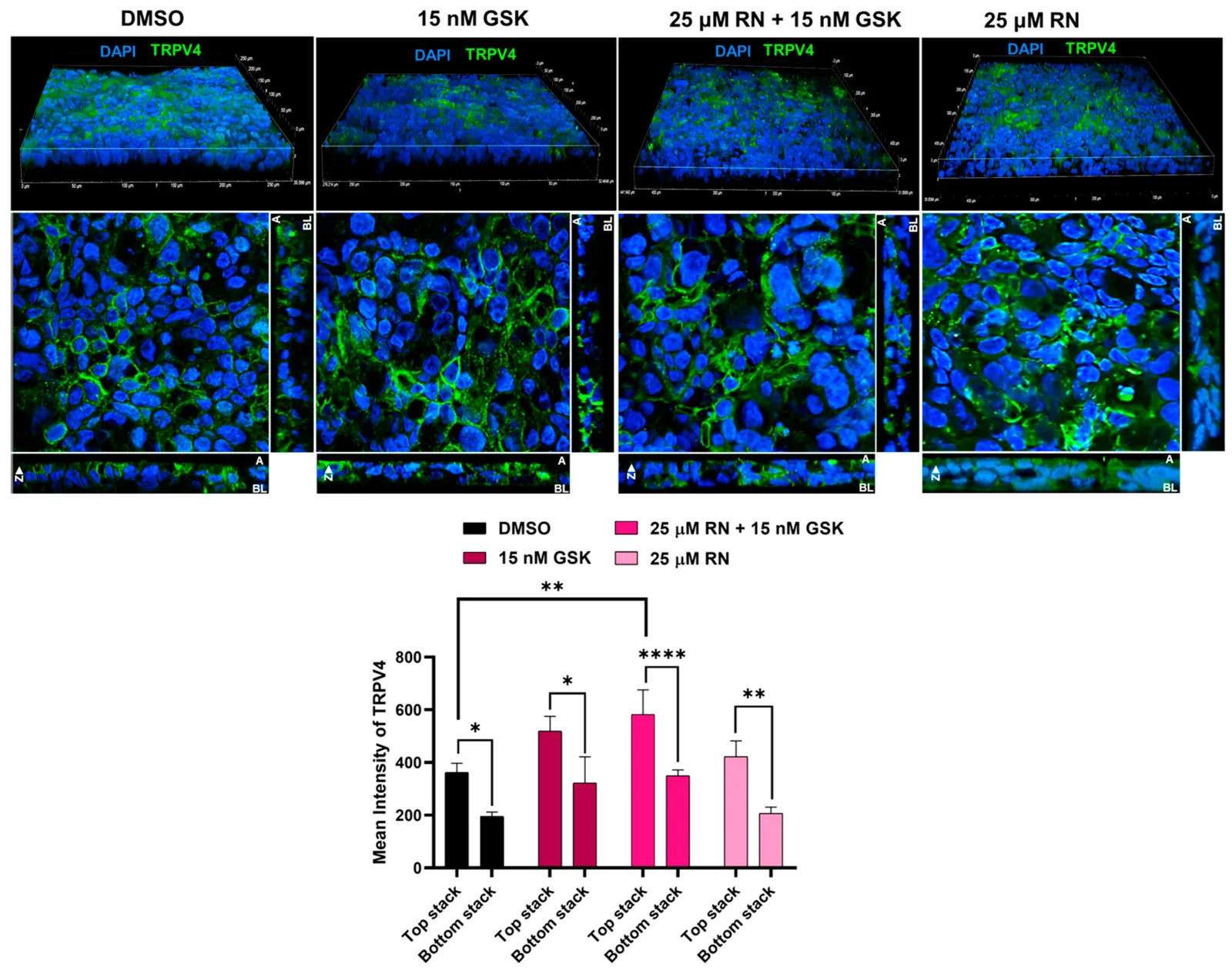
TRPV4 localization in HIBCPP cells: The effect of TRPV4 activation on TRPV4 localization was examined by immunofluorescence. Maximum-intensity projections and three-dimensional (3D) xz-plane renderings of cells grown on permeable supports coated with Collagen-IV and stained for TRPV4 (green) and DAPI (blue) are shown under the following conditions: DMSO vehicle control; 15 nM GSK1016790A for 10 min; and 25 µM RN1734 for 10 min followed by 15 nM GSK1016790A for 10 min; and 25 µM RN1734 for 10 min. Z-stack images were quantified on four quarters. The top and bottom 25 slices were selected to represent the apical and basolateral regions, respectively, for comparative analysis of TRPV4 immunoreactivity. Statistical significance was assessed using a two-tailed unpaired Student’s *t*-test comparing each treatment condition with the corresponding DMSO control stack (top or bottom, as applicable) (*n* = 3) (* *p* value < 0.033, ** *p* > 0.002, **** *p* > 0.0001).

Next, we investigated the localization of TRPV4 in CPe cells using immunocytochemistry studies to assess its expression and distribution upon activation and inhibition. Previous studies have shown that TRPV4 is predominantly localized to the apical membrane of CPe cells [10,46]. In our study, we sought to examine whether TRPV4 localization is affected by its activation and/or inhibition. An immunocytochemistry study was performed under four experimental conditions, with TRPV4 expression probed using an anti-TRPV4 antibody. TRPV4 localization remained predominantly apical (top stack) following treatment with the TRPV4 agonist, the antagonist, and the antagonist-followed-by-agonist combination, although a modest fraction of intracellular signal was observed in the bottom stack, closest to the basolateral membrane (Figure 5). TRPV4 immunofluorescence intensity was significantly increased in the top stack of GSK1016790A-treated cells compared with the DMSO control, while no significant difference was observed in the bottom stack. Total TRPV4 protein levels remained unchanged (Figure 4B). Therefore, the increased apical signal may reflect changes in TRPV4 localization or antibody epitope accessibility following TRPV4 activation. Although the bottom stack showed a smaller fraction of overall TRPV4 mean intensity, no statistically significant differences were observed between the bottom stacks of the treatment groups relative to the DMSO control. Therefore, the increased apical signal may reflect changes in TRPV4 localization, clustering, internalization, or antibody epitope accessibility following TRPV4 activation.

### 2.3. Pharmacological Modulation of AMPK-Associated Signaling Alters TRPV4-Mediated Barrier Permeability

One of the key candidates identified from our phosphoproteomics study was AMPK. AMPK activity is canonically regulated by phosphorylation of Thr172 within the activation loop of the catalytic α subunit. Additional phosphorylation sites, including Ser496 on AMPKα1, have been reported to negatively modulate AMPK signaling under specific cellular contexts. In our phosphoproteomic analysis, AMPKα1 phosphorylation was detected at Ser496 following GSK1016790A treatment (Table 1). Given that Ser496 phosphorylation may represent a regulatory event downstream of TRPV4 activation, we sought to examine the functional effects associated with pharmacological modulation of AMPK-related signaling. Additionally, TRPV4 activation was associated with increased phosphorylation of the TJ proteins ZO-1 and CLDN7 at several serine residues.

**Figure 6:**
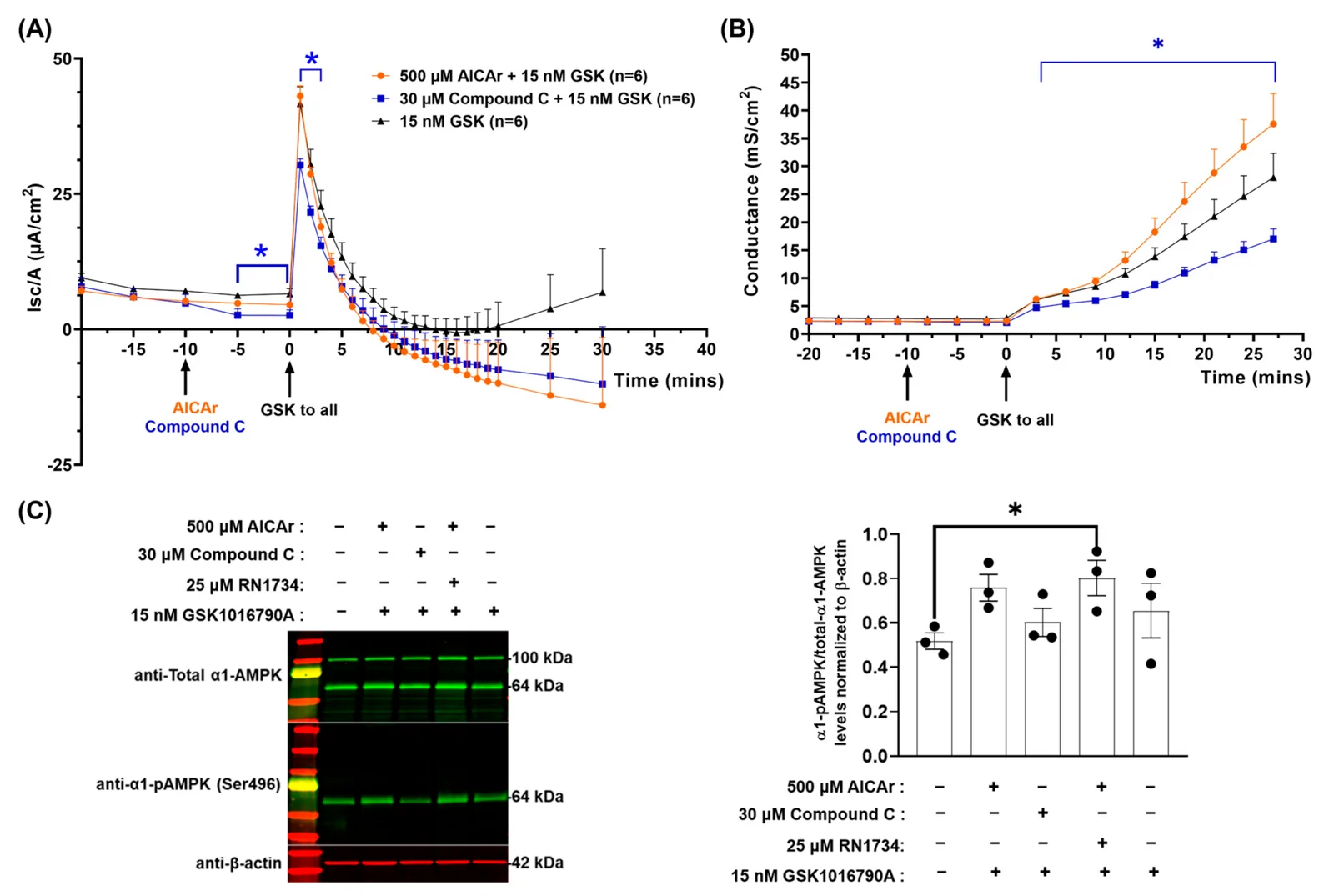
Pharmacological modulation of AMPK-associated signaling alters TRPV4-mediated effects on epithelial permeability: (**A**) Changes in the short-circuit current (I_sc_) over time were measured by electrophysiology after treatment with AICAr followed by GSK1016790A (dark orange), Compound C followed by GSK1016790A (blue), and GSK1016790A alone (black). (**B**) Changes in membrane permeability, measured as conductance (mS/cm^2^), were monitored over time after treatment with AICAr followed by GSK1016790A (dark orange), Compound C followed by GSK1016790A (blue), and GSK1016790A alone (black). AICAR, Compound C, and GSK1016790A were added to the apical side only. GSK1016790A was applied to all conditions at t = 0 min, AICAr and Compound C were added at t = −10 min. The response to GSK1016790A was monitored for 30 min from t = 0, while responses to the other drugs were monitored for 10 min. Arrows indicate the time the drugs were applied. Multiple Student’s *t*-tests were performed in GraphPad Prism 8.0.1. Traces represent mean ± SD from six biological replicates (*n* = 6), and the significance is indicated relative to the GSK1016790A control for each condition (* *p* < 0.05). (**C**) HIBCPP cells untreated and treated with AICAr + GSK1016790A, Compound C + GSK1016790A, AICAr + GSK1016790A + RN1734, and GSK1016790A alone were analyzed for total α1-AMPK and α1-pAMPK (Ser496) levels by Western blot (**left panel**). β-actin was used as the housekeeping protein for normalization. Quantification of the blots is shown in the right panel. Individual blot bands for phosphorylated AMPK (α1-pAMPK) to total α1-AMPK levels were normalized to their respective β-actin levels. All values represent the mean ± standard deviation of three biological replicates (*n* = 3). Statistical significance was assessed using a two-tailed unpaired Student’s *t*-test. No significant differences were observed in the α1-pAMPK to total α1-AMPK levels amongst the groups except for AICAr + GSK1016790A + RN1734 vs. untreated group (* *p* value < 0.033).

To further investigate the functional consequences of TRPV4-mediated AMPK modulation on epithelial barrier function, we performed Ussing chamber electrophysiological analyses to assess how TRPV4 activation influences AMPK activity, ion transport, and epithelial barrier function. AMPK-associated signaling was pharmacologically modulated using AICAr and Compound C, followed by TRPV4 agonist stimulation. Short-circuit current (I_sc_; a measure of net transepithelial electrolyte flux and, transepithelial conductance; a measure of permeability or barrier integrity) were monitored under the following conditions: (1) 500 µM AICAr + 15 nM GSK1016790A, (2) 30 µM Compound C + 15 nM GSK1016790A, and (3) 15 nM GSK1016790A alone. Compound C had an inhibitory effect on the basal level of electrolyte flux across the epithelium before the addition of the TRPV4 agonist. Net electrogenic ion transport (I_sc_) stimulated in response to TRPV4 activation was not significantly altered across experimental groups with the exception of a Compound C-induced inhibition of transepithelial ion flux in the very early time points (1–2 min) of the GSK1016790A response (Figure 6A). Conversely, the TRPV4-stimulated increase in membrane conductance was markedly affected by AMPK modulation. Neither the AMPK activator nor inhibitor had an effect on basal membrane permeability. However, pre-treatment with the AMPK activator, AICAr, appeared to potentiate the TRPV4 mediated electrolyte flux (although was not statistically significant). In contrast, pretreatment with Compound C, the ATP-competitive inhibitor of the AMPK catalytic domain, reduced the TRPV4-stimulated transepithelial conductance significantly, suggesting enhanced barrier integrity/decreased permeability (Figure 6B).

Furthermore, we validated the protein levels of total α1-AMPK and α1-pAMPK (Ser496) in HIBCPP cells upon AMPK modulation. We utilized the same concentrations and incubation times on cells in electrophysiological studies (except that GSK1016790A treatment was limited to 10 min for the Western blot experiments) and probed with total and phospho (Ser496) AMPK antibodies (Table S1). The α1-pAMPK (Ser496)-to-total α1-AMPK ratio varied between treatment groups. In particular, activation of AMPK active form with AICAr followed by GSK1016790A and AICAr followed by GSK1016790A and RN1734 resulted in increased α1-pAMPK to total α1-AMPK ratio when compared to untreated group, suggesting induction of inhibitory AMPK phosphorylation upon AICAr + GSK1016790A and AICAr + GSK1016790A + RN1734 treatment. In contrast, inhibition of AMPK inhibitory form with Compound C followed by GSK1016790A reduced α1-pAMPK to total α1-AMPK levels relative to the AICAr group (although not statistically significant). This finding is consistent with previous studies reporting that Compound C inhibits AMPK kinase activity, thereby reducing Ser496 autophosphorylation [28]. Together with our electrophysiological findings, these results support the notion that reduced inhibitory phosphorylation of AMPK is associated with enhanced barrier function (Figure 6C). These biochemical findings align with our functional data, where elevated and sustained inhibitory phosphorylation of AMPKα1 at Ser496 following AICAr + GSK1016790A treatment likely triggers feedback inhibition of AMPK activity, leading to disrupted epithelial barrier function and increased permeability [28]. Conversely, Compound C treatment followed by GSK1016790A reduced Ser496 phosphorylation and corresponded with decreased permeability. Together, these results support a model in which TRPV4 activation engages AMPK signaling at Ser496 to regulate epithelial barrier properties, such that inhibitory phosphorylation of AMPK compromises barrier integrity, and conversely, reduced inhibitory phosphorylation preserves barrier function.

### 2.4. AICAr-Induced ST-Loop Phosphorylation Coincides with Increased Epithelial Barrier Discontinuity Following TRPV4 Activation

**Figure 7:**
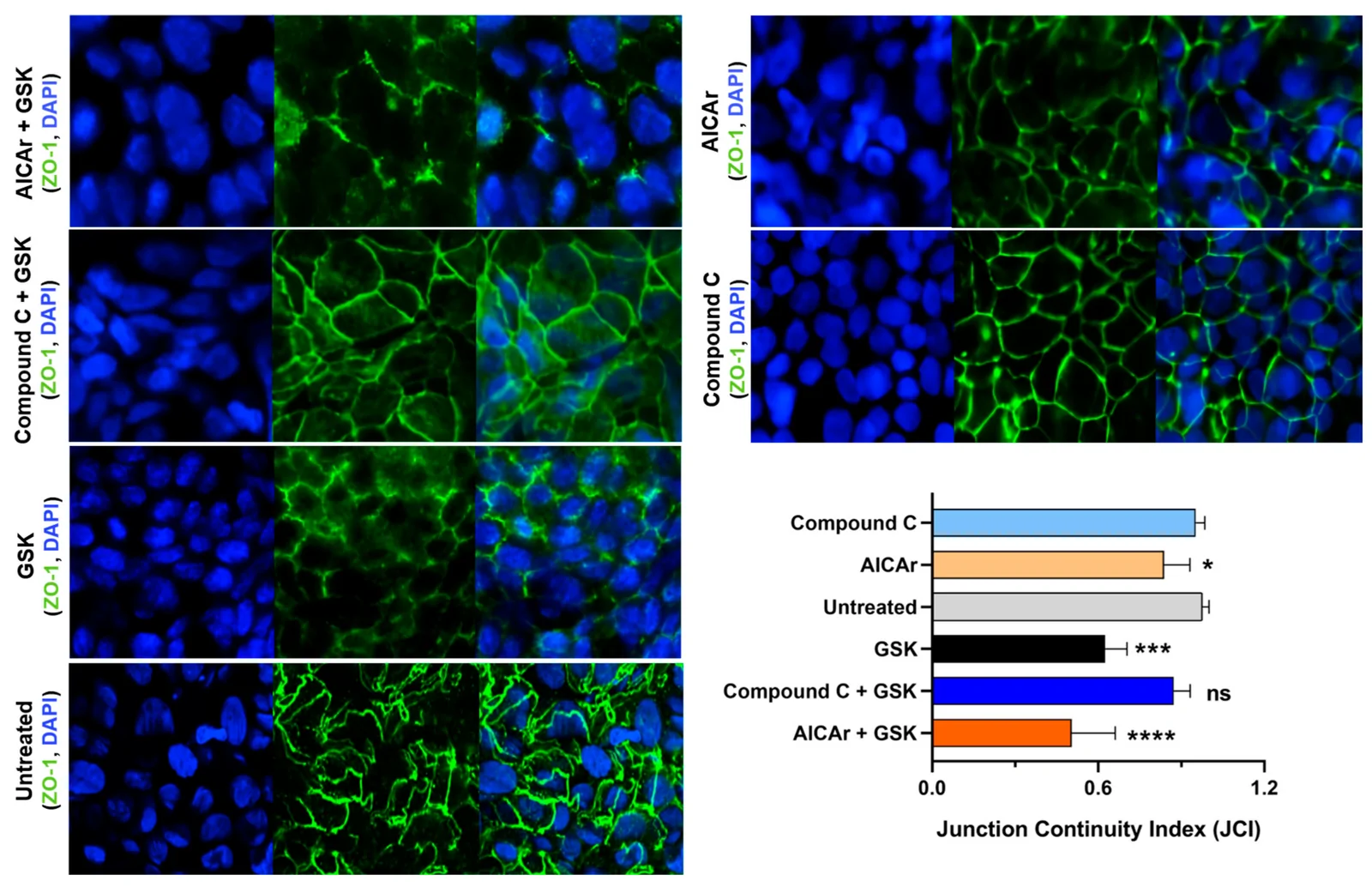
AMPK modulates the TJ integrity: Epithelial membrane integrity was assessed by treating the cells with AMPK and TRPV4 pharmacological modulators (AICAr+GSK1016790A, Compound C+GSK1016790A, GSK1016790A, AICAr, Compound C) and without any treatment (untreated) in an immunocytochemistry study. Untreated cells were used as control. ZO-1 (green) expression was quantified to calculate the junction continuity index (JCI). Statistical significance was assessed using a two-tailed unpaired Student’s *t*-test against the untreated group. Untreated cells were used as the reference control for this imaging experiment. A separate DMSO vehicle-control group was not included and therefore, comparisons with the untreated group do not control for potential vehicle-associated effects. Images are representative of three biological replicates (*n* = 3) (^ns^ *p* value < 0.1234, * *p* value < 0.033, *** *p* > 0.0002, **** *p* > 0.0001).

In order to further classify the potential roles of AMPK in maintaining barrier permeability, we performed immunofluorescence studies followed by the treatments with AICAr + GSK1016790A, Compound C + GSK1016790A, GSK1016790A alone, AICAr alone, and Compound C alone and compared it against untreated controls. We monitored the TJ expression of ZO-1 and quantified the junction continuity index (JCI), which measures the degree of fragmentation of the barrier with respect to the drug treatments. Upon treatment with GSK1016790A, the ZO-1 expression appeared discontinuous with a lower JCI than the untreated controls. Interestingly, AICAr treatment followed by GSK1016790A enhanced this more, further decreasing the JCI significantly compared to the untreated controls, indicative of more disrupted and less organized ZO-1 expression (Figure 7). In contrast, upon Compound C treatment with GSK1016790A, the barrier integrity was preserved similar to the untreated controls. Compound C treatment alone still maintained the barrier integrity with continuous ZO-1 expression as shown with high JCI values (Figure 7). Overall, the ZO-1 expression patterns observed following pharmacological modulation were consistent with an association between AMPK-related signaling and TRPV4-dependent changes in TJ integrity, although AMPK activity was not directly measured in these experiments.

### 2.5. Metformin Pretreatment Decreases TRPV4-Induced Barrier Permeability, Independent of ST-Loop Phosphorylation Status

**Figure 8:**
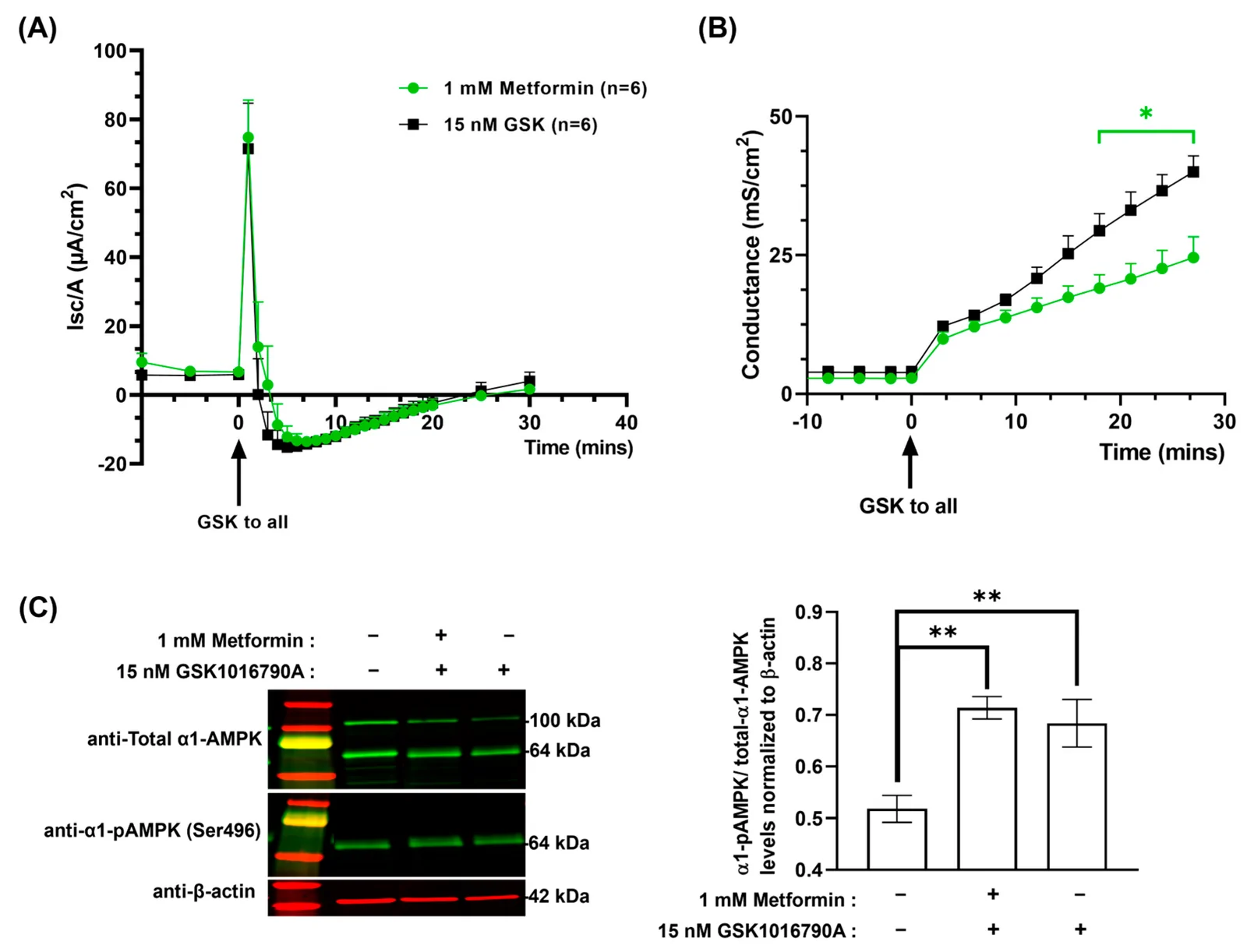
Metformin pretreatment alters TRPV4-mediated transepithelial permeability: (**A**) Changes in the short-circuit current (I_sc_) over time and (**B**) Changes in membrane permeability, measured as conductance (mS/cm^2^), were measured by electrophysiology after treatment with 1 mM metformin for 24 h followed by GSK1016790A (green), and GSK1016790A alone (black). Cells were exposed to Metformin for 24 h prior to the experiment. GSK1016790A was added to the apical side of both conditions at t = 0. Multiple Student’s *t*-tests were performed in GraphPad Prism 8.0.1 (* *p* < 0.05). Traces represent mean ± SD from six biological replicates (*n* = 6), and the significance is indicated relative to the GSK1016790A control for each condition. (**C**) HIBCPP cells untreated and treated with Metformin + GSK1016790A, and GSK1016790A alone were analyzed for total α1-AMPK and α1-pAMPK (Ser496) levels by Western blot (**left panel**). β-actin was used as the housekeeping protein for normalization. Quantification of the blots is shown in the right panel. Individual blot bands for phosphorylated AMPK (α1-pAMPK) to total α1-AMPK ratio were normalized to their respective β-actin levels. All values represent the mean ± SD of three biological replicates (*n* = 3). Statistical significance was assessed using a two-tailed unpaired Student’s *t*-test against the untreated control (** *p* value < 0.0021).

Given the regulatory role of AMPK in epithelial barrier function that we are reporting in this study, we used metformin, a well-characterized anti-diabetic drug and an AMPK activator, to evaluate whether the modulation of this pathway with metformin influences the observed effects. The cells were treated with 1 mM metformin for 24 h at 37 °C followed by TRPV4 activation to measure the transepithelial net electrogenic ion movements and changes to the permeability. Our electrophysiological data showed no significant changes in TRPV4-stimulated transepithelial ion flux (I_sc_) (Figure 8A) but a significant decrease in TRPV4-mediated transepithelial permeability upon TRPV4 agonist treatment (Figure 8B), indicating that metformin pretreatment was associated with enhanced TJ integrity and decreased barrier permeability following TRPV4 stimulation.

Furthermore, total α1-AMPK and α1-pAMPK (Ser496) levels were quantified with metformin + GSK1016790A and GSK1016790A control and compared against untreated controls. Metformin-treated cells with GSK1016790A, and GSK1016790A alone group showed higher α1-pAMPK to total α1-AMPK ratio than the untreated controls (Figure 8C). As metformin is primarily known to exert its effects through AMPK-dependent signaling pathways, and our electrophysiological data demonstrated reduced epithelial permeability following metformin and GSK1016790A treatment, lower levels of inhibitory AMPK phosphorylation at Ser496 would have been anticipated. Contrary to this expectation, Ser496 phosphorylation was increased. Several mechanisms may explain the observed increase in Ser496 phosphorylation. One possibility is that prolonged metformin exposure resulted in sustained AMPK activation, leading to Ser496 autophosphorylation as a negative-feedback mechanism that limits AMPK activity to some extent. Alternatively, Ser496 phosphorylation may be mediated by another upstream kinase independent of the canonical cAMP/PKA pathway, as Akt has also been reported to phosphorylate this inhibitory residue [47]. These possibilities suggest that multiple regulatory pathways may contribute to the modulation of AMPK activity under our experimental conditions, which will require further investigation.

## 3. Discussion

The barrier function of the CPe is regulated through highly specialized epithelial transporters, ion channels, and TJ complexes, enabling tight control of the production and composition of CSF. Through coordinated ion transport, water flux, and selective permeability, CPe plays a central role in maintaining CSF homeostasis and intracranial pressure, processes that become dysregulated in conditions such as hydrocephalus. In this context, studies have highlighted the importance of mechanosensitive ion channels in modulating CSF dynamics, particularly through their influence on epithelial transport and barrier properties [10,48]. Among these, TRPV4 has been implicated as a key regulator of osmosensation and mechanotransduction in CPe, where it contributes to cellular responses to mechanical and osmotic stress. Importantly, a previous in vivo study has demonstrated that TRPV4 inhibition with a TRPV4 antagonist can ameliorate ventriculomegaly in hydrocephalic rat models, underscoring its functional relevance in regulating CSF production and ventricular expansion [16]. However, the downstream mechanisms by which TRPV4 mediates these effects remain incompletely understood, particularly in respect to intracellular signaling pathways that link TRPV4-mediated ion flux to epithelial barrier regulation and CSF homeostasis. To address this gap, we investigated TRPV4-dependent signaling using a phosphoproteome-wide approach in a human choroid plexus epithelial cell model (HIBCPP cells) with short-term TRPV4 agonist treatment, TRPV4 antagonist pretreatment followed by agonist stimulation, and TRPV4 antagonist alone conditions, enabling systematic assessment of phosphorylation changes associated with both activation and inhibition of TRPV4 signaling. Our analysis identified a subset of differentially phosphorylated proteins that were consistently altered upon TRPV4 modulation, with functional enrichment primarily in AMP kinase-associated signaling networks and TJ-related proteins (ZO-1 and CLDN7) (Figure 2), demonstrating that TRPV4 activation triggers coordinated downstream kinase signaling cascades that converge on regulators of epithelial junctional architecture, indicating a potential mechanism by which mechanosensitive signaling may influence TJ integrity and, in turn, contribute to the regulation of CSF dynamics in hydrocephalus.

AMPK is recognized as a central energy sensor and a regulator of epithelial barrier integrity, with multiple studies signifying its role in maintaining TJ stability in both epithelial and endothelial systems [31,40,49]. More importantly, AMPK regulates the barrier function through phosphorylation of junctional and cytoskeletal proteins, including components of the apical junctional complexes such as ZO proteins and claudins, thereby promoting TJ assembly and preserving barrier permeability under metabolic or mechanical stress conditions [50]. Consistent with the established barrier-supportive functions of AMPK, our findings demonstrated an increase in an inhibitory phosphorylation site of AMPK (Ser496) upon GSK1016790A treatment (Figure 2, GSK1016790A vs. DMSO), suggesting a potential attenuation of AMPK signaling in response to TRPV4 stimulation, raising the possibility that TRPV4-mediated signaling may counteract AMPK-dependent barrier-supportive pathways in CPe. Our study also demonstrated AMPK modulation with AICAr followed by GSK1016790A treatment, which resulted in increased epithelial permeability (although not statistically significant), and increased AMPKα1 Ser496 phosphorylation while Compound C treatment with GSK1016790A reversed these effects (Figure 6B). Our electrophysiological and biochemical data were consistent with alterations in barrier integrity following pharmacological modulation and TRPV4 stimulation.

Furthermore, our JCI measurements of ZO-1 expression supported our electrophysiological findings under AMPK and TRPV4 modulation. ZO-1 expression showed increased discontinuity following TRPV4 stimulation with GSK1016790A, which was further enhanced with AICAr + GSK1016790A treatment, indicative of a potential contribution of inhibitory AMPK phosphorylation (Ser496) to compromised barrier integrity. In line with this, increased Ser496 phosphorylation under AICAr + TRPV4-stimulated conditions (Figure 6C) was associated with increased barrier leakiness, as reflected by altered ZO-1 organization. Because AMPK kinase activity was not directly measured, the functional consequence of Ser496 phosphorylation on AMPK activity under these conditions remains inferential. Although Hulme and colleagues reported increased conductance following TRPV4 activation without overt disruption of ZO-1 spatial organization, our quantitative JCI analysis detected decreased continuity of junctional ZO-1 staining. This does not necessarily indicate complete junctional disassembly, but rather a more subtle alteration in ZO-1 continuity that may accompany the increased conductance observed following TRPV4 activation. Differences in the assessment of overall spatial organization versus quantitative junctional continuity may therefore contribute to the apparent difference between the studies.

In contrast, Compound C + TRPV4 stimulation resulted in more continuous ZO-1 expression, with JCI values almost similar to the untreated controls (~1.0). Because Compound C inhibits AMPK activation, including phosphorylation at Thr172, reduced AMPK activity may also limit downstream inhibitory autophosphorylation at Ser496. We therefore propose that reduced Ser496 phosphorylation may contribute to the more continuous ZO-1 expression and preservation of barrier integrity observed following Compound C treatment, consistent with our electrophysiological findings (Figure 7).

**Figure 9:**
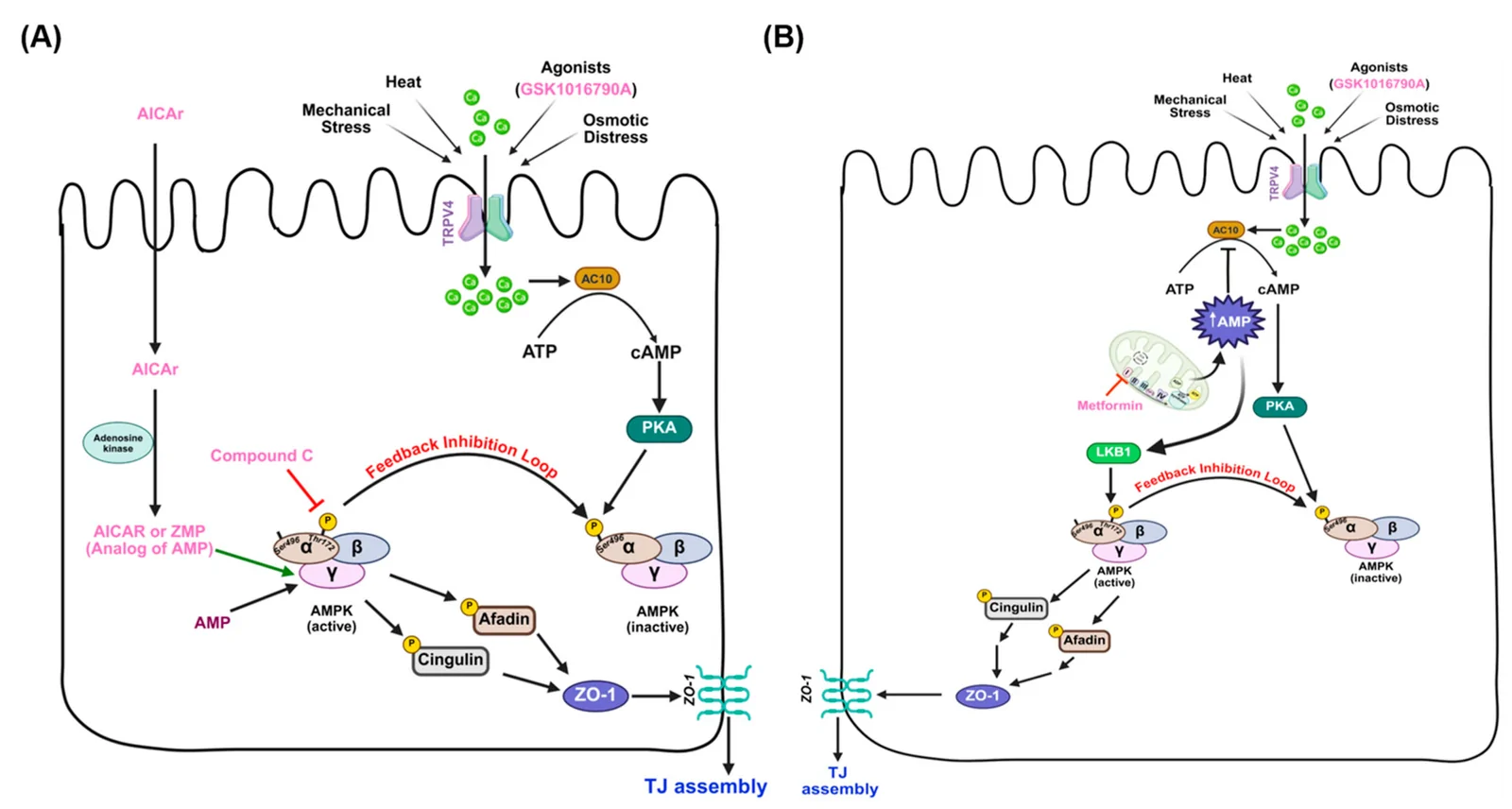
Schematic created in BioRender illustrating the mechanism of action of (**A**) AICAr, Compound C and (**B**) Metformin under cellular conditions. Pathways downstream of TRPV4 activation are shown. Protein activation is represented in black arrows, while inhibition is shown in red with blunt-end arrows. Phosphorylation sites reported in the literature and/or observed in our study are indicated (Thr172 and Ser496 in AMPK). Note: AICAr is the cell-permeable nucleoside form of the compound. Following cellular uptake, AICAr is phosphorylated by adenosine kinase to AICAR (also known as ZMP), an AMP analog that binds the AMPK γ subunit and activates AMPK. AC10, adenylate cyclase 10; LKB1, liver kinase B1; PKA, protein kinase A; cAMP, cyclic adenosine monophosphate; ZO-1, zonula occludens-1. Possible drugs that target each pathway are shown in pink.

However, AICAr or Compound C treatment alone did not significantly affect barrier integrity, whereas TRPV4 stimulation with GSK1016790A following AICAr or Compound C disrupted ZO-1 continuity, strongly suggesting that AMPK-mediated regulation of TJ integrity is dependent on TRPV4 modulation. This finding would explain the apparent paradox in these studies. Notably, AICAr is classically an AMPK activator, and Compound C is an AMPK inhibitor. Therefore, if the mechanism of action was simply AMPK activation, one would expect that AICAr would tighten the junctional complexes and decrease transepithelial conductance while Compound C would do the converse. This is opposite of what we find in our electrophysiology and immunocytochemistry experiments. Clearly TRPV4 activation plays a modulating role, likely by changing the conformation/phosphorylation of AMPK and, thereby, altering the final effect. One possible explanation is that AICAr activates AMPK through its intracellular conversion to the AMP analog (AICAR or ZMP), after its phosphorylation by adenosine kinases and directly binds to AMPK at its γ-subunit. Based on previous studies, this is expected to promote Thr172 phosphorylation and AMPK activation, while sustained AMPK activation has been proposed to promote Ser496 phosphorylation as a negative-feedback mechanism. In contrast, Compound C inhibits AMPK catalytic activity by directly binding to the catalytic site and would therefore be expected to reduce Ser496 autophosphorylation (Figure 9A). Thus, the increased Ser496 phosphorylation observed following AICAr treatment and its reduction following Compound C treatment along with TRPV4 agonist are consistent with Ser496 functioning as a feedback regulatory site rather than the initiating mechanism of AMPK activation. Although we feel this is the most likely scenario, other explanations are possible because both AICAr and Compound C have off-target effects.

We further note that our biochemical readout of AMPK status throughout this manuscript is limited to pSer496 phosphorylation normalized to total AMPKα1, and we propose three future directions to further define the relationship between AMPK phosphorylation status and functional activity. First, directly measuring Thr172 phosphorylation would be useful for confirming that the Ser496 changes we report actually track AMPK activation. Second, measuring phosphorylation of the downstream substrate ACC at Ser79 would be useful for obtaining a substrate-level, ST-loop-independent readout of AMPK catalytic output, confirming whether each pharmacological treatment actually altered AMPK signaling reaching its substrates. Third, an in vitro AMPK kinase activity assay on immunoprecipitates would be useful for testing catalytic activity directly, establishing whether the phosphorylation changes we observe reflect a genuine change in enzymatic activity rather than antibody-detectable phosphorylation state alone.

Given the apparent interplay between AMPK and TRPV4 signaling, we sought to determine whether other pharmacologically available modulators of AMPK would result in similar effects on epithelial barrier function. The emerging literature highlights the role of metformin, a widely used FDA-approved anti-diabetic drug, as an activator of the AMPK pathway [51]. In epithelial systems, metformin-mediated AMPK activation has been associated with enhanced barrier function, whereas inhibition of AMPK attenuates these protective effects, supporting a functional AMPK dependence [52,53]. Studies imply that metformin may activate AMPK, further reinforcing its role to probe AMPK-dependent signaling [54,55]. Given this, our study also explored the effects of metformin in relation to AMPK-associated signaling, by performing electrophysiological measurements of net electrogenic ion movement and conductance, to further investigate the AMPK pathway involvement. Although metformin treatment followed by TRPV4 agonist did not alter net transcellular ion movement, conductance was significantly reduced, suggesting enhanced barrier integrity following metformin pretreatment (Figure 8B) independent of transepithelial ion flux. It should also be noted that the 1 mM metformin concentration used in this in vitro study exceeds the plasma concentrations typically achieved clinically, which are generally in the micromolar range. Furthermore, metformin exposure within the CNS/CSF is limited and may differ substantially from that achieved in cultured cells. Therefore, the present metformin experiments should be interpreted as pharmacological interrogation of barrier-associated signaling rather than evidence supporting metformin as a therapeutic intervention for hydrocephalus. Thus, the observed reduction in TRPV4-stimulated conductance following metformin treatment provides a basis for further investigation at clinically relevant concentrations and in models that allow direct measurement of choroid plexus fluid secretion.

The decrease in conductance observed with metformin treatment is consistent with activation of the LKB1–AMPK pathway in response to increased AMP levels, reflecting a cellular energy stress signal. Under these conditions, AMPK activation may shift signaling away from cAMP/PKA-dependent pathways. Upon TRPV4 activation, which elevates intracellular Ca^2+^, this balance between AMPK and cAMP/PKA signaling may contribute to the observed changes in epithelial barrier function as illustrated by the pathway diagram (Figure 9B). As LKB1 phosphorylates AMPK at Thr172 residue, it keeps AMPK in its active form and thereby promotes the TJ integrity and reduced permeability [56]. Although the activation of the LKB1-AMPK pathway can indirectly suppress cAMP/PKA signaling which could prevent the inhibitory phosphorylation on Ser496, our biochemical data show higher Ser496 in metformin and GSK1016790A treated conditions. This may imply that alternative signaling pathways may be engaged under these conditions in addition to its feedback inhibition loop. One potential explanation is the activation of compensatory kinases, such as Akt (PKB) [29], which are also capable of mediating Ser496 phosphorylation. These findings point to a more complex regulatory network and warrant further investigation to understand the signaling mechanisms involved.

It is worth noting that AMPK activators produced opposite effects on barrier permeability in this study. This divergence reflects their distinct upstream mechanisms of AMPK activation. AICAr is converted intracellularly to ZMP which binds the AMPK γ-subunit and acutely promotes LKB1-mediated Thr172 phosphorylation within minutes [57]. Metformin, by contrast, activates AMPK only indirectly. At the concentration and duration used in this study (1 mM, 24 h), it primarily inhibits mitochondrial complex I, lowering cellular ATP and raising the AMP/ADP:ATP ratio, and can act through a separate, largely AMP-independent lysosomal pathway involving Axin1-scaffolded LKB1–AMPK [58,59]. Metformin’s primary effect by inhibiting complex I independently limits the ATP available to the ion transporters (Na^+^/K^+^-ATPase, NKCC1) that drive transepithelial conductance in CPe cells [1,60]. The 24 h exposure also gives time for compensatory changes in TJ trafficking that the acute 10 min AICAr treatment cannot develop. Thus, metformin’s barrier-tightening effect likely reflects this bioenergetic and lysosomal route, rather than the same acute Thr172/Ser496 axis engaged by AICAr, even though both compounds ultimately increase ST-loop phosphorylation.

An important limitation of this study is that the functional assessment of AMPK-associated signaling relied on pharmacological modulation. Compound C, AICAr, and metformin are also known to exert AMPK-independent effects. Therefore, the observed changes in transepithelial conductance and junctional organization cannot be attributed exclusively to AMPK activity. Although PRKAA1 (AMPKα1) was independently identified by phosphoproteomic analysis following TRPV4 activation, the present findings support an association between TRPV4 activity, AMPK-associated signaling, and epithelial barrier regulation rather than establishing AMPK-specific causality. Future studies using genetic approaches, such as PRKAA1/PRKAA2 knock-down or manipulation of the AMPKα1 Ser496 phosphorylation site, will be required to establish the specific contribution of AMPK to the TRPV4-dependent barrier response. An additional limitation is the use of HIBCPP cells, which are derived from a human choroid plexus papilloma and therefore may not fully recapitulate the physiology of normal choroid plexus epithelium. Nevertheless, HIBCPP cells represent one of the few well-characterized human choroid plexus epithelial models available and retain key blood–CSF barrier characteristics, making them a useful model for investigating epithelial signaling and barrier regulation.

In the present study, reduced conductance is consistent with increased epithelial barrier tightness; yet conductance measurements alone cannot distinguish between paracellular and transcellular contributions. However, whether increasing choroid plexus epithelial barrier tightness would reduce or otherwise beneficially modify CSF production in hydrocephalus needs further investigation. CSF secretion depends on coordinated transcellular ion and water transport in addition to paracellular permeability, and we did not directly measure CSF secretion. Therefore, the direction of barrier modulation that would be therapeutically beneficial in hydrocephalus remains to be established in appropriate physiological and in vivo models.

Overall, our findings identify an association between TRPV4 activity, AMPK-associated signaling, and epithelial barrier regulation in HIBCPP cells. These results provide a framework for further investigation of how TRPV4-associated signaling influences choroid plexus epithelial function. Integrating our electrophysiological, biochemical, and imaging data, we propose this mechanistic model (Figure 9A,B) in which TRPV4 activation converges on AMPK signaling to regulate epithelial barrier properties, highlighting potential points for pharmacological intervention (given in pink).

## 4. Materials and Methods

### 4.1. Collagen-IV Coating of the Transwell Insert Filters

Cells were grown on Transwell membrane supports that were collagen coated. A concentration of 2 mg/mL stock solution of Collagen-IV (Sigma, St. Louis, MO, USA, Cat No: C5533) was prepared by dissolving the powder in ice-cold 0.25% acetic acid. The 2 mg/mL stock solution was diluted in a ratio of 1:40 with sterile MilliQ water in a conical tube to obtain a final working solution of 50 µg/mL. A volume of 300 µL of the working solution was added to the apical side (top) of each insert, followed by gentle shaking or tapping of the plate to ensure that the entire filter was evenly covered by the coating solution. Transwell plates were placed in the 37 °C, 5% CO_2_ incubator for 1 h. After 1 h, the coating solution was carefully aspirated, and the filters were air dried for approximately 20 min under sterile conditions before the cells were seeded onto the Transwells.

### 4.2. Cell Culture

HIBCPP cells, a well-characterized model of choroid plexus epithelial cells derived from a female cancer patient were used in this study [22]. Cells were cultured in 25-cm^2^ flasks in Dulbecco’s Modified Eagle Medium (DMEM powder, Gibco, Waltham, MA, USA, Cat No: 12100-046: High Glucose + L-Glutamine + Phenol Red w/3.7 g/L Bicarbonate) supplemented with 10% fetal bovine serum (Gemini Bio Benchmark, West Sacramento, CA, USA, Cat No: 100-106), 1% antibiotic (Penicillin/Streptomycin 100×, Cytiva, Marlborough, MA, USA, Cat No: SV30010), 0.125% insulin (Human recombinant 4 mg/mL in zinc solution, Gibco, Cat No: 12585-014), and 3.7 g/L sodium bicarbonate (Fisher Scientific, Waltham, MA USA, Cat No: S233-500) hereafter referred to as “DMEM complete media”. Media in 25-cm^2^ flasks were replenished every other day until the cells reached ~90% confluency at 37 °C and 5% CO_2_. Cells were detached from flasks using 0.25% sterile-filtered Trypsin-EDTA (Sigma Aldrich, Cat No: T4049) after two washes with Hank’s balanced salt solution (HBSS, Seattle, WA, USA, Gibco, Cat No: 14170-112) for 5 min. After that, the cells were spun down, resuspended in 5 mL per flask, and reseeded onto the 25-cm^2^ flasks to continue until seven passages from the time of thawing and into collagen-coated 6-well Transwell plates (2 mL per insert) for further experiments. The Transwell membrane inserts used in these experiments were Costar 0.4 µm polycarbonate membrane tissue culture treated (Corning, Corning, NY, USA, Cat No: 3412) and coated with Collagen-IV as described in Section 4.1. Cells seeded onto the Transwell inserts were cultured for 12–16 days, with DMEM complete media for the first 8 days and DMEM serum-free media (DMEM powder, 1% antibiotic (Penicillin/Streptomycin 100×), and 0.125% insulin (Human recombinant 4 mg/mL in zinc solution), and 1.85 g/L sodium bicarbonate) thereafter. Media replenishment for the Transwell until day 8 was done every other day with DMEM complete media and after day 8, cells were fed with DMEM serum-free media every day.

### 4.3. Protein Extraction

HIBCPP cells cultured on the Costar Transwell inserts without collagen coating were used in protein extraction studies. Parallel cultures of cells were tested using Ussing chamber electrophysiology to make sure the TEER value is above 300 Ω·cm^2^ before proceeding with protein extraction. Transwell media were changed to fresh DMEM serum-free media 1 h before treatment of the cells. After 1 h, cells were treated with vehicle (DMSO) or relevant drugs (TRPV4 agonist: GSK1016790A, TRPV4 antagonist: RN1734). Four experimental conditions used in this study for phosphoproteomics mass spectrometry and Western blot were (1) DMSO treatment for 10 min, (2) 15 nM GSK1016790A treatment for 10 min, (3) 25 µM RN1734 treatment for 10 min followed by 15 nM GSK1016790A treatment for 10 min, and (4) 25 µM RN1734 treatment for 10 min. The 10 min treatment period was selected to capture rapid phosphorylation-dependent signaling events following TRPV4 modulation while minimizing longer-term cellular responses. This time course correlated with TRPV4-mediated changes in transepithelial electrolyte flux as well as TRPV4-mediated changes in transepithelial permeability. For each treatment condition, cells from three wells were pooled to obtain sufficient protein for analysis. Each pooled sample constituted one biological replicate, and three independently prepared pooled samples were analyzed per condition (*n* = 3). After treatment, cells were washed with HBSS for 5 min twice. Cells were then scraped with fresh HBSS and centrifuged at 1200 rpm (145 rcf) for 5 min. Cell pellets were resuspended in 1× RIPA lysis buffer (Millipore Sigma, Burlington, MA, USA, Cat No: 20-188) with Halt protease inhibitor cocktail (Thermo Scientific Cat No: 78441). Dissolved pellets were incubated on ice for 30 min followed by brief sonication for 7 s, 3 times with 30 s intervals on ice. After sonication, cells were centrifuged at 12,000 rpm (14,400 rcf) for 20 min at 4 °C and the supernatant was collected and saved as extracted protein from each condition. Protein concentration was quantified using a DC assay kit (Bio-Rad, Hercules, CA, USA, Cat No: 5000114) with bovine serum albumin (BSA, Bio-Rad, Cat No: 5000206) as standard according to the manufacturer’s protocol [61]. All conditions were performed in triplicate (*n* = 3).

Western blots were performed using protein extracts obtained from five experimental conditions: (1) untreated cells, (2) treatment with 500 µM AICAR for 10 min followed by 15 nM GSK1016790A for 10 min; (3) treatment with 30 µM Compound C for 10 min followed by 15 nM GSK1016790A for 10 min; (4) treatment with 500 µM AICAR for 10 min followed by 15 nM GSK1016790A for 10 min and subsequently 25 µM RN1734 for 10 min; and (5) treatment with 15 nM GSK1016790A for 10 min alone. All conditions were performed in triplicate (*n* = 3). Protein concentrations were determined using a DC Protein Assay Kit (Bio-Rad) with BSA as the standard, according to the manufacturer’s instructions.

### 4.4. Global and Phosphoproteomics Mass Spectrometry

Methods described below are adaptations from literature reports [62] and vendor-provided protocols. Samples (*n* = 3 replicates of 4 conditions) were precipitated using trichloroacetic acid (TCA, 20% vol/vol) overnight at 4 °C followed by centrifugation at 12,000 rcf for 30 min and washing of the pellets twice with ice-cold acetone. Proteins were suspended in 8 M urea with 100 mM Tris pH 8.5 and protein concentrations were determined by Bradford protein assay (Bio-Rad, Cat No: 5000006). Approximately 60 µg equivalent of protein from each sample was then reduced with 5 mM tris(2-carboxyethyl) phosphine hydrochloride (TCEP, Sigma-Aldrich, Cat No: C4706) for 30 min at room temperature and alkylated with 10 mM chloroacetamide (CAA, Sigma Aldrich, Cat No: C0267) for 30 min at room temperature in the dark. Samples were diluted with 50 mM Tris HCl, pH 8.5 to a final urea concentration of 2 M for Trypsin/Lys-C based overnight protein digestion at 35 °C (a 1:50 protease-to-substrate ratio, mass Spectrometry-grade, Promega Corporation, Madison, WI, USA, Cat No: V5072.) Digestions were acidified with trifluoroacetic acid (TFA, 0.5% *v*/*v*) and desalted on Sep-Pak^®^ Vac cartridges (Waters^TM^, Milford, MA, USA, Cat No: WAT054955) with a wash of 1 mL 0.1% TFA followed by elution in 70% acetonitrile containing 0.1% formic acid (FA).

TMT labeling: All peptide samples (*n* = 3 replicates of 4 conditions) were each labeled with Tandem Mass Tag (TMTpro) reagent per manufacturer’s instructions (0.5 mg per sample; ThermoFisher Scientific, TMTpro™ Isobaric Label Reagent Set, Milford, MA, USA, Cat No: A44520 Lot ZJ405903) overnight at room temperature. Samples were checked to confirm >95% labeling efficiency and then quenched with a final concentration *v*/*v* of 0.3% hydroxylamine at room temperature for 15 min. Labeled peptides were then mixed and dried by speed vacuum. Excess TMTpro reagent was removed from the mix by desalting on a 50 mg Waters Seppak cartridge as described above.

Phosphopeptide Enrichment: For phosphoproteomics, the combined, labeled peptide mixture was then applied to a Pierce High-Select™ TiO_2_ Phosphopeptide enrichment tip (ThermoFisher Scientific, Cat No: A32993). After preparing spin tips as per manufacturer’s instructions, the sample was applied twice, washed, and eluted as per manufacturer’s instructions. The phosphopeptide elution was immediately dried and then resuspended in 25 µL buffer A. For global proteomics, the flowthrough from the phosphopeptide enrichment step was dried down by speed vacuum, reconstituted in 0.1% TFA, and one third was fractionated on Sep-Pak^®^ Vac cartridges using methodology and reagents from Pierce™ High pH reversed-phase peptide fractionation kit (Fractions with 12.5%, 15%, 17.5%, 22%, 22.5%, 25%, 35%, and 70% acetonitrile in 0.1% triethylamine, ThermoFisher Cat No: 84868).

Samples were run (1/8th of each global fraction and 1/5th followed by a tech rep of 1/3rd of each phosphopeptide fraction) on an EASY-nLC 1200 HPLC system (SCR: 014993, ThermoFisher Scientific) coupled to an Eclipse Orbitrap™ mass spectrometer with FAIMSpro interface (ThermoFisher). Each multiplex was run on a 25 cm Aurora Ultimate TS column (Ion Opticks Cat No: AUR3-25075C18) in a 50 °C column oven with a 180 min gradient. The gradient was run from 8 to 38%B over 160 min; 30–95% B over 10 min; held at 80% for 2 min; and dropping from 95 to 5% B over the final 5 min (Mobile phases A: 0.1% formic acid (FA), water; B: 0.1% FA, 80% acetonitrile (ThermoFisher, Cat No: LS122500)). The mass spectrometer was operated in positive ion mode, default charge state of 2, advanced peak determination on, and Easy IC™ on. Three FAIMS CVs were utilized (−45 CV; −55 CV; −65 CV and a technical replicate with −40 CV, −50 CV, and −60 CV) each with a cycle time of 1 s and identical MS and MS2 parameters. Precursor scans (*m*/*z* 400–1600) were done with an Orbitrap resolution of 120,000, RF lens% 30, 50 ms maximum inject time, standard automatic gain control (AGC) target, minimum MS2 intensity threshold of 2.5 × 10^4^, MIPS mode to peptide, including charges of 2 to 6 for fragmentation with 60 s dynamic exclusion shared across the cycles, excluding isotopes. MS2 scans were performed with a quadrupole isolation window of 0.7 *m*/*z*, 32% HCD collision energy, 50,000 resolution, 200% AGC target, dynamic maximum IT, fixed first mass of 100 *m*/*z*.

Data analysis: Resulting RAW files were analyzed in Proteome Discoverer™ 2.5.0.400 (ThermoFisher) with a Homo sapiens UniProt reviewed proteome FASTA (downloaded 051322, 20292 sequences) plus common laboratory contaminants (73 sequences). The database search was performed using the reviewed canonical human UniProt protein sequences; alternative isoform sequences were not included as separate entries in the database search. SEQUEST HT searches were conducted with full trypsin digest, 3 maximum number missed cleavages; precursor mass tolerance of 10 ppm; and a fragment mass tolerance of 0.02 Da. Static modifications used for the search were: (1) TMTpro label on peptide N-termini, (2) TMTpro label on lysine (K), and carbamidomethylation on cysteine (C) residues. Dynamic modifications used for the search were (1) oxidation on methionine (M) residues, (2) phosphorylation on serine, threonine or tyrosine residues (S, T, Y), (3) deamidation of asparagine or glutamine (N,Q), (4) acetylation on protein N-termini, (5) methionine loss on protein N-termini or (6) acetylation with methionine loss on protein N-termini. A maximum of 3 dynamic modifications were allowed per peptide. Percolator False Discovery Rate (FDR) was set to a strict setting of 0.01 and a relaxed setting of 0.05. IMP-ptm-RS node was used for all modification site localization scores. Values from both unique and razor peptides were used for quantification. Proteins were identified based on peptide-spectrum matches generated by SEQUEST HT and filtered using Percolator. Proteins sharing identified peptides were grouped, with a master protein assigned by Proteome Discoverer to represent each protein group. In the consensus workflows, peptides were normalized to total peptide amount. Quantification methods utilized TMTpro isotopic impurity levels available from ThermoFisher. Reporter ion quantification filters were set to an average S/N threshold of 6 and a co-isolation threshold of 30%. Modified peptides were excluded from protein-level quantification, normalization and roll-up. Resulting grouped abundance values for each sample type, abundance ratio values, and respective unadjusted *p*-values (calculated by Protein Abundance ratio with no imputation and ANOVA (individual protein)) from Proteome Discoverer (ThermoFisher, PD 2.5) were exported to Microsoft Excel. The *p*-values used for differential phosphorylation comparisons were nominal (unadjusted) and were not corrected for multiple testing. Therefore, these analyses are considered exploratory and were used to identify candidate phosphorylation changes for subsequent investigation. Accordingly, phosphorylation events meeting the predefined thresholds were considered exploratory candidate features rather than false-discovery rate (FDR)-significant phosphoproteome-wide discoveries. Processed data files for Global and Phosphoproteomics are provided in Supplementary Table S1.

### 4.5. Artificial Cerebrospinal Fluid (aCSF) Preparation

Bicarbonate-free aCSF was used in the electrophysiological chambers and on cells 1.5 h prior to experimentation. aCSF was made fresh daily by mixing solution A (17.3 g/L NaCl, 0.45 g/L KCl, 0.41 g/L CaCl_2·_2H_2_O, 0.33 g/L MgCl_2·_6H_2_O) and solution B (0.43 g/L Na_2_HPO_4·_7H_2_O, 0.055 g/L NaH_2_PO_4·_H_2_O) made in MilliQ water, mixed in a 1:1 ratio [63]. The solution was adjusted to pH 7.1 using 5M NaOH. After bubbling fresh aCSF with CO_2_ for 15–20 min, culture medium from the Transwell inserts was removed and replaced with fresh aCSF apically and serum-free DMEM basolaterally. Cells were incubated at 37 °C and 5% CO_2_ for 1.5 h before electrophysiology or subsequent analyses.

### 4.6. Electrophysiological Studies

An eight chamber Ussing chamber electrophysiology instrument (Physiologic Instruments Inc., Venice, FL, USA) was used to measure transepithelial electrical resistance (TEER). The inverse of TEER is conductance, which reflects the overall electrical permeability of the epithelium but does not distinguish between paracellular and transcellular conductive pathways. Simultaneous changes in short-circuit current (I_sc_) were measured, reflecting net electrogenic ion transport across the epithelium. Cells grown on collagen-coated membrane inserts were used in the electrophysiology experiments. Cells were tested in the electrophysiology system between days 12 and 16 after seeding onto the membrane inserts, and only those exhibiting TEER values above 300 Ω·cm^2^ were used. Before starting the experiments, cell media were replenished with freshly prepared aCSF on the apical side and DMEM serum-free media on the basolateral side and incubated for 1.5 h at 37 °C and 5% CO_2_. Before measuring cell-containing inserts, background resistance of the system was blanked using inserts with only the membrane (no cells). For measurements, Transwell inserts containing confluent monolayers of cultured cells were placed between the chamber halves (P2315A sliders using chamber slide with the area of 1.26 cm^2^), and electrodes were placed at their respective ports. The apical and basolateral sides of the chambers were filled with pre-warmed, oxygenated fresh aCSF and the chambers were equilibrated at 37 °C, using a temperature-controlled water bath. Chambers were continuously aerated with 95% O_2_ and 5% CO_2_. Electrodes from the Ussing chamber were connected to the multichannel voltage/current clamp controller and calibration was performed. To compensate for system asymmetries, resistance readings from the blank membranes were recorded and later subtracted from the measurements obtained with cell-containing inserts by the system. The tissues were allowed to equilibrate for 10 min prior to experimental recording in the system. Short-circuit current (I_sc_; µA), electrical resistance (TEER; Ohm.cm^2^) and conductance were recorded by the system. The AQUIRE 3.0 data acquisition program (Physiologic Instruments Inc.), connected to the electrophysiology system, was used to calculate the TEER (Rt × A), short-circuit current normalized to area of the Transwell insert (I_sc_/A), and conductance normalized to area (Gt/A), where A represents the surface area of the cell monolayer. Rt was determined from brief current pulses. Resistance values were normalized to the surface area and expressed in Ω·cm^2^. Only TEER values above 300 Ω·cm^2^ were considered for further experimentation. Baseline I_sc_ was monitored continuously, and drugs (AICAR, Compound C, GSK1016790A, and RN1734) were added to the apical side for the required time periods (Refer to the drug treatment groups below). Data were collected and analyzed with AQUIRE 3.0 software.

### 4.7. Drug Treatment Groups Used in Electrophysiological Studies

Once the TEER value of the cells were confirmed (>300 Ω·cm^2^), cultures were allowed to stabilize for 10 min before adding any drugs in the system. The effect of AMPK on TRPV4 activity was assessed by treating cells on the apical side under the following conditions: 500 µM AMPK activator (AICAR; Millipore Sigma, Burlington, MA, USA, Cat No: A9978) for 10 min followed by 15 nM TRPV4 agonist (GSK1016790A; Millipore Sigma, Cat No: 942206-85-1) for 30 min; or 30 µM AMPK inhibitor (Compound C; Millipore Sigma, Cat No: 171260) for 10 min followed by 15 nM GSK1016790A for 30 min. To assess the impact of TRPV4 inhibition on AMPK-mediated modulation, cells were treated with 500 µM AICAR for 10 min, and 15 nM GSK1016790A for 30 min. As a control, to evaluate the baseline TRPV4 agonist effects, cells were treated with 15 nM GSK1016790A alone for 30 min.

For the metformin study, the cells were treated with 1 mM of metformin for 24 h in the 37 °C incubator. 15 nM of GSK1016790A was added when the cells were mounted in the electrophysiological system the following day. GSK1016790A alone was added to the control groups. All electrophysiological experiments were performed using six independent biological replicates (*n* = 6), with each replicate representing an independently prepared cell monolayer. Statistical significance was evaluated using multiple Student’s *t*-tests in GraphPad Prism 8.0.1.

### 4.8. Western Blot

Total protein extracted from each experimental condition (30 µg) was loaded onto the 4–15% Mini-PROTEAN TGX gels (Bio-Rad, Cat No: 456-8086) with precision plus protein dual color standards ladder (Bio-Rad, Cat No: 1610374) at 120 V for 2 h at room temperature. Gels were run in 1× Tris/Glycine/SDS buffer (Bio-Rad, Cat No: 610732). Following electrophoretic separation, proteins were transferred onto nitrocellulose membranes using ice-cold 1× Trans-Blot Turbo transfer packs (Bio-Rad, Cat No: 1704157) in Trans-Blot Turbo transfer apparatus operated with the preconfigured mixed–molecular weight setting (Bio-Rad, Cat No: 1704150). Membrane transfer efficiency and total protein content were checked by staining with Ponceau S (0.1% Ponceau S in 5% glacial acetic acid, ThermoFisher, Cat No: A40000279). Membranes were blocked in 1× Tris-buffered saline (TBS, Bio-Rad, Cat No: 1706435) containing 5% non-fat dry milk, followed by overnight incubation at 4 °C with gentle shaking in the desired primary antibodies diluted in blocking buffer supplemented with 0.01% Tween-20 (1× TBST, Bio-Rad, Cat No: 1610781). The following day, membranes were washed three times for 5 min in 1× TBST and then incubated with secondary antibodies diluted in 5% milk in 1× TBST for 1 h (See Tables S1 and S2 for primary and secondary antibody details, respectively). Final washes were performed in 1× TBST, and immunoreactive signals were captured using a Li-Cor Odyssey CLx imaging system. Quantitative analysis of band intensities was done with Image Studio Lite™ image processing software (LI-COR, RRID:SCR_013715). For band intensity normalization, β-actin was used as a loading control utilizing the same antibody incubation protocol. All experimental conditions were performed in biological triplicate (*n* = 3).

### 4.9. RT-PCR

RNA was isolated from cells cultured as confluent monolayers in 25 cm^2^ flasks using the Monarch Total RNA Miniprep Kit (NEB, Ipswich, MA, USA, Cat No: T2010S). RNA concentration was determined using a Nanodrop 2000 spectrophotometer (ThermoFisher) before cDNA synthesis. cDNA was synthesized from total RNA using LunaScript RT SuperMix Kit (NEB, Cat No: E3010L) and subsequently used for PCR analyses. Primer pairs designed using Primer-BLAST [64] and those obtained from the literature [10] were used in this RT-PCR study. New primer pairs were ordered from Integrated DNA Technologies. Details of the primers used in this study are given in Table S3. PCR was performed with an annealing temperature of 62 °C on all primers after optimization. Following PCR, electrophoresis was performed with a 2% agarose gel stained with SYBRSafe Gel Stain (Invitrogen, Carlsbad, CA, USA, Cat No: S33102) for *TRPV4*, *ZO-1*, *Claudin-7* and *GAPDH* genes along with no-template control and no reverse transcriptase control. Bands were visualized under UV light with a ChemiDoc touch imaging system (Bio-Rad, version 3.0.1).

### 4.10. Immunocytochemistry

Cells grown on collagen-coated Transwell inserts were used for immunocytochemistry. Cells were used after confirming their barrier integrity (TEER > 300 Ω·cm^2^). Media were changed to aCSF on top and DMEM serum-free on the bottom 1.5 h prior to drug treatments. Following the drug treatments, cells were washed with 1× phosphate-buffered saline (PBS) twice and fixed in ice-cold 4% paraformaldehyde (PFA) in 1× PBS for 10 min. Following fixation, cultures were rinsed three times with 1× PBS (5 min per wash). The filters were then cut and sectioned into four pieces and transferred to 12-well plates for immunostaining. Membranes were permeabilized for 10 min in buffer containing 0.2% Triton X-100, 3% goat serum, and 1% BSA in 1× PBS, followed by a 30 min blocking step using 3% goat serum and 1% BSA in 1× PBS. Membranes were subsequently incubated with primary antibodies (anti-TRPV4; Abcam, Cambridge, UK, Cat No: AB314454 at 1:250 dilution, and anti-ZO1; Abcam, Cat No: AB216880 at 1:250 dilution) diluted in blocking buffer overnight at 4 °C with gentle agitation. On the following day, membranes were rinsed three times with 1× PBS (5 min per wash) and then incubated with secondary antibodies (goat anti-rabbit IgG (H+L) Alexa Fluor Plus 488; Invitrogen, Cat No: A32731TR) prepared in blocking buffer (1:1000 dilution) for 1 h at room temperature in the dark. Samples were washed thrice with 1× PBS (5 min each) and subsequently stained with DAPI nuclear dye (0.5 µg/mL in 1× PBS) for 10 min at room temperature, protected from light. After a final 5 min wash with 1× PBS, membranes were mounted onto slides using Aqua Poly-Mount (Polysciences Aqua-Polymount, Warrington, PA, USA, Cat No: 18606-5) and were sealed with coverslips. Imaging was performed using a Nikon AX confocal system (Nikon Corporation, Tokyo, Japan) to acquire 40X water lens (40X Apo LWD Lambda S) and Z-stack images. No-antibody controls were utilized to confirm the nonspecific bindings from the secondary antibodies. All experimental conditions were performed in biological triplicate (*n* = 3). All confocal images were processed in NIS-Elements AR version 5.30.04.

ZO-1 junctional continuity was quantified from confocal fluorescence images using ImageJ software (1.54t). For each biological replicate, images were acquired from representative fields of the ZO-1-stained epithelial monolayer under identical acquisition settings across treatment groups. Images were converted to grayscale and subjected to the same background subtraction and thresholding procedure for all experimental groups. A binary mask was generated using a consistent threshold applied across all images, and the ZO-1-positive junctional structures were analyzed to quantify junctional continuity. The JCI was calculated as the ratio of continuous ZO-1-positive junctional signal to the total junctional signal within the analyzed region, with higher JCI values indicating more continuous and organized junctional staining and lower values indicating greater junctional discontinuity/fragmentation. Three non-overlapping fields were analyzed per biological replicate, with three independent biological replicates (*n* = 3) for each treatment condition. Junctional ZO-1 staining across the entire field was included in the JCI analysis rather than selecting individual cells as independent observations. Image acquisition, processing, thresholding, and quantification parameters were kept consistent across all groups.

### 4.11. Statistical Analysis

Quantitative data are shown as the mean ± standard deviation from three independent experiments (*n* = 3), except for electrophysiological studies (*n* = 6). Statistical significance was assessed using either a two-tailed unpaired Student’s *t*-test or a two-way ANOVA, performed in GraphPad Prism 8.0.1 (RRID: SCR_002798). Significance thresholds were set at *p* ≤ 0.05, with the following notation: **** *p* < 0.0001, *** *p* < 0.0002, ** *p* < 0.0021, * *p* < 0.033, and ^ns^ *p* ≥ 0.1234.

## Figures and Tables

**Table 1:** Candidate phosphoproteins and phosphorylation sites identified in the exploratory phosphoproteomic analysis.

Phosphoprotein	UniProt Accession Number	Phospho Residues
CLDN7	O95471	S204, S206, S207
PRKAA1	Q13131	S508, S486, S496
ZO-1	G3V1L9	S422, S416, S264, S1710, S427 (Phosphosite numbering corresponds to the UniProt accession assigned during the Proteome Discoverer search. ZO-1/TJP1 phosphosites are reported according to accession G3V1L9.)

## Data Availability

The mass spectrometry proteomics and phosphoproteomics data generated in this study have been deposited in the ProteomeXchange Consortium via the PRIDE partner repository under the dataset identifier, PXD083736. All other data supporting the findings of this study are available within the article and its Supplementary Materials. The study utilized the previously established HIBCPP human choroid plexus papilloma cell line originally described by Ishiwata et al. (2005) and obtained for this study from Profs. Horst Schroten and Christian Schwerk from the Department of Pediatrics, Medical Faculty Mannheim, Heidelberg University under a Material Transfer Agreement.

## Supplementary Materials

The following supporting information can be downloaded at: https://www.mdpi.com/article/10.3390/ijms27188305/s1.
