# Supplementary material for: Phosphoproteomic Insights into TRPV4–AMPK Signaling Axis and Barrier Regulation in the Choroid Plexus Epithelium
Source: Int J Mol Sci. 2026 Sep 18;27(18):8305. doi: 10.3390/ijms27188305 (PMC13607985; doi:10.3390/ijms27188305)
Supplement: Supplementary file 1 [file ijms-27-08305-s001.zip › IJMS_SI_AMPK MANUSCRIPT.pdf]

International Journal of Molecular Sciences

MDPI

Article

Phosphoproteomic Insights into TRPV4–AMPK Signaling Axis and Barrier Regulation in the Choroid Plexus Epithelium

Gowthami Mahendran[1], Maryam Torabi[1], Bonnie Blazer-Yost[1]*

[1] Hydrocephalus Research Center, Department of Biology, Indiana University, Indianapolis, Indiana, USA
* Correspondence: bblazer@iu.edu

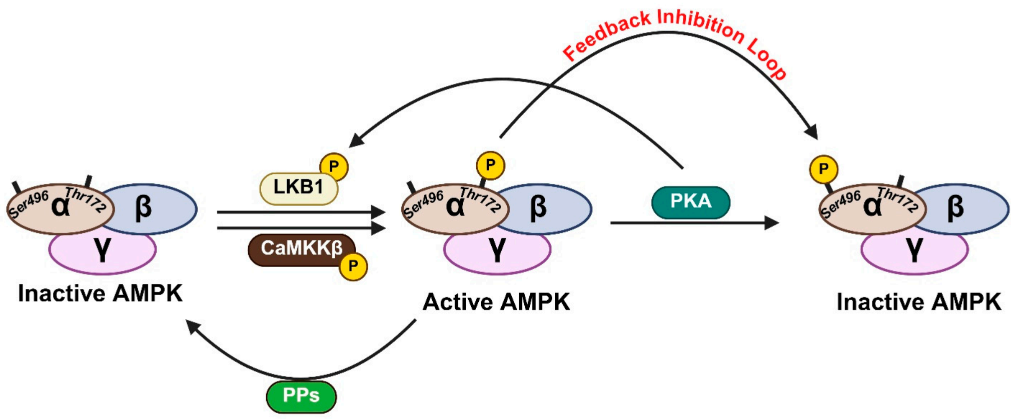

Figure. S1 | Dynamic regulation of AMPK activity: AMPK activation is mediated by phosphorylation of Thr172 on the α subunit by upstream kinases LKB1 and CaMKKβ, whereas dephosphorylation by protein phosphatases (PPs) and inhibitory regulation by protein kinase A (PKA) promote AMPK inactivation while activated AMPK also phosphorylates at Ser496 through autophosphorylation to result in feedback inhibition. These opposing mechanisms tightly control AMPK activity in response to cellular signaling and metabolic status.

Table S1: Primary antibodies used in western blot studies

Primary antibody	Species and clonality	Manufacturer	Antibody dilution
TRPV4	Rabbit recombinant multiclonal	Abcam (AB314454)	1:1000
AMPK	Rabbit polyclonal	Abcam (AB3759)	1:1000
p-AMPK (S496)	Rabbit monoclonal	Abcam (AB92701)	1:1000
β-actin	Rabbit polyclonal	Proteintech (20536-1-AP)	1:1000

Table S2: Secondary antibodies used in western blot studies

Secondary antibody	Manufacturer	Antibody dilution

Donkey anti-rabbit IgG (H+L) (Alexa Fluor-790)	Jackson Immunoresearch (711-655-152)	1:4000
Donkey anti-mouse IgG (H+L) (Alexa Fluor-680)	Jackson Immunoresearch (715-625-150)	1:4000

Table S3: Primer sequences used in RT-PCR study

Gene Name	Forward primer (5'-3')	Reverse primer (5'-3')	Product size (bp)
TRPV4	AAGTTCAAGGACTGGGCC-TATG	ACGATCACCAGGACAGAG-TAGA	473
AQP1	AGATCAGCATCTTCCGTG	AGTTGTGTGTGATCACCG	354
TJP1 (ZO-1)	GAGGAC-CAGCTGAAGGACAG	GGCCACTTCTTGGATCATGT	243
CLDN7 (Claudin-7)	CTCGAGCCCTAATGGTGGTC	GCAAGGAGATCCCAGGTCAC	414
GAPDH	AAGCCTCAAGATCATCAG-CAA	CCCTGTTGCTGTAGCCAAATTC	546
